# High Electron Mobility Thin‐Film Transistors Based on Solution‐Processed Semiconducting Metal Oxide Heterojunctions and Quasi‐Superlattices

**DOI:** 10.1002/advs.201500058

**Published:** 2015-05-26

**Authors:** Yen‐Hung Lin, Hendrik Faber, John G. Labram, Emmanuel Stratakis, Labrini Sygellou, Emmanuel Kymakis, Nikolaos A. Hastas, Ruipeng Li, Kui Zhao, Aram Amassian, Neil D. Treat, Martyn McLachlan, Thomas D. Anthopoulos

**Affiliations:** ^1^ Department of Physics and Centre for Plastic ElectronicsBlackett LaboratoryImperial College LondonLondonSW7 2AZUK; ^2^ Dutch Polymer Institute (DPI)P.O. Box 9025600AXEindhovenThe Netherlands; ^3^ Institute of Electronic Structure and Laser (IESL)Foundation for Research and Technology‐Hellas (FORTH)Heraklion71003Greece; ^4^ Materials Science & Technology DepartmentUniversity of CreteHeraklion71003Greece; ^5^ Institute of Chemical Engineering and High Temperature Processes (ICEHT)Foundation of Research and Technology Hellas (FORTH)Stadiou Strasse PlataniP.O. Box 1414PatrasGR‐265 04Greece; ^6^ Center of Materials Technology and Photonics and Electrical Engineering DepartmentTechnological Educational Institute (TEI) of CreteHeraklion71004Greece; ^7^ Physics DepartmentAristotle University of ThessalonikiThessaloniki54124Greece; ^8^ Cornell High Energy Synchrotron SourceWilson LaboratoryCornell UniversityIthacaNY14853USA; ^9^ Materials Science and EngineeringDivision of Physical Sciences and EngineeringKing Abdullah University of Science and TechnologyThuwal23955–6900Saudi Arabia; ^10^ Department of Materials and Centre for Plastic ElectronicsImperial College LondonLondon Royal School of MinesLondonSW7 2AZUK

**Keywords:** energy quantization, metal oxides, solution‐processed materials, superlattices, transistors, transparent electronics

## Abstract

High mobility thin‐film transistor technologies that can be implemented using simple and inexpensive fabrication methods are in great demand because of their applicability in a wide range of emerging optoelectronics. Here, a novel concept of thin‐film transistors is reported that exploits the enhanced electron transport properties of low‐dimensional polycrystalline heterojunctions and quasi‐superlattices (QSLs) consisting of alternating layers of In_2_O_3_, Ga_2_O_3,_ and ZnO grown by sequential spin casting of different precursors in air at low temperatures (180–200 °C). Optimized prototype QSL transistors exhibit band‐like transport with electron mobilities approximately a tenfold greater (25–45 cm^2^ V^−1^ s^−1^) than single oxide devices (typically 2–5 cm^2^ V^−1^ s^−1^). Based on temperature‐dependent electron transport and capacitance‐voltage measurements, it is argued that the enhanced performance arises from the presence of quasi 2D electron gas‐like systems formed at the carefully engineered oxide heterointerfaces. The QSL transistor concept proposed here can in principle extend to a range of other oxide material systems and deposition methods (sputtering, atomic layer deposition, spray pyrolysis, roll‐to‐roll, etc.) and can be seen as an extremely promising technology for application in next‐generation large area optoelectronics such as ultrahigh definition optical displays and large‐area microelectronics where high performance is a key requirement.

## 1 Introduction

Thin‐film transistors (TFTs) based on transparent metal oxide semiconductors represent an emerging technology that promises to revolutionize large‐area electronics due to the high carrier mobility,^1^ optical transparency,^2^ mechanical flexibility,^3^ and the potential for low‐temperature processing.^4^ Like many other transistor technologies, the performance level of oxide TFTs ultimately depends on the intrinsic properties of the semiconducting material employed.^5^ As a result, the maximum electron mobility that can be achieved in conventional devices is limited by the intrinsic mobility of the semiconductor used. In the case of a handful of inorganic transistor technologies (e.g., GaN,^6^ GaAs,^7^), this intrinsic mobility limitation has been overcome through the use of epitaxially grown low‐dimensional heterostructures composed of an undoped (intrinsic) layer and an extrinsically doped semiconducting layer.^8^, ^9^ In such heterostructures, the majority carriers minimize their energy by diffusing out of the doped semiconductor layer and into the lower potential undoped semiconductor where they form a 2D electron gas (2DEG) in close proximity to the heterointerface.^10^, ^11^ A key aspect of the 2DEG systems is that the confined carriers become spatially separated from the donor/acceptor sites leading to a reduction in ionized impurity scattering and to high charge carrier mobilities, which in many cases exceed the bulk mobility of the individual semiconductors used.^8^, ^9^, ^10^, ^11^

Recently, there has been a mounting interest in 2DEG systems formed at epitaxially grown insulating metal oxide heterointerfaces^12^ due to the very high charge carrier mobilities^13^ and their potential for high‐performance electronics^14^, ^15^ as well as the rich new physics.^16^ Building on the early work on insulating oxides, Tampo et al.^17^ demonstrated the formation of 2DEGs in semiconducting ZnO/MgZnO heterointerfaces for which electron mobilities exceeding 700 000 cm^2^ V^−1^ s^−1^, albeit at cryogenic temperatures, have recently been reported.^18^

A more technologically relevant development was the recent implementation of the ZnO/MgZnO heterointerface as the active channel in high mobility TFTs.^19^ The latter work can be seen as the steppingstone towards practical application of the oxide 2DEG technology in large‐area thin‐film electronics.^20^, ^21^, ^22^ A summary of the field‐effect electron mobility values reported in recent years for different metal oxide heterointerface systems grown by different methods (e.g., sputtering, molecular beam epitaxy (MBE), metalorganic chemical vapor deposition (MOCVD), and atomic layer deposition (ALD) techniques) is given in Table S1 (Supporting Information). Despite these very promising early results and the tremendous potential of the 2DEG transistor technology, however, its widespread adoption in practical electronic applications is currently hampered by the rather complex^23^ and high temperature (600–900 °C, see Table S1, Supporting Information) manufacturing processes often required in order to ensure the formation of the all‐important high‐quality heterointerface.^19^, ^24^, ^25^ Because of the latter requirement it is not a trivial question whether high‐quality oxide heterointerfaces can be realized using simpler, cost‐efficient, and high‐throughput fabrication methods that are compatible with existing semiconductor fabrication processes (e.g., solution‐based) and even perhaps temperature‐sensitive substrate materials such as plastic. Thus, the development of ease to implement metal oxide hetero/multilayer structures could help overcoming important bottlenecks associated with the level of performance and manufacturing of incumbent TFT technologies, and enable the emergence of a host of large‐area, flexible optoelectronics.^4^, ^26^, ^27^, ^28^, ^29^, ^30^

Here, we report the development of low‐dimensional quasi‐superlattices (QSLs) grown by sequential deposition of different metal oxides by spin casting and thermal annealing at temperatures in the range of 180–200 °C. Structural characterization of the QSLs revealed the existence of discrete binary oxide layers and the presence of high‐quality heterointerfaces. Remarkably, we found that when the QSLs are incorporated as the active channels in TFTs, the electron mobility is enhanced by approximately one order of magnitude and the charge transport becomes temperature independent resembling band‐like conduction. The incorporation of solution‐processed QSLs as active channels in TFTs not only substantially improves the electrical performance of the devices but also brings a new perspective on the design principles that can be used to develop the next‐generation metal oxide semiconductors, devices, and circuits.

## 2 Results

### 2.1 Growth and Structural Characterization of Low‐Dimensional Metal Oxide Layers

**Figure 1:**
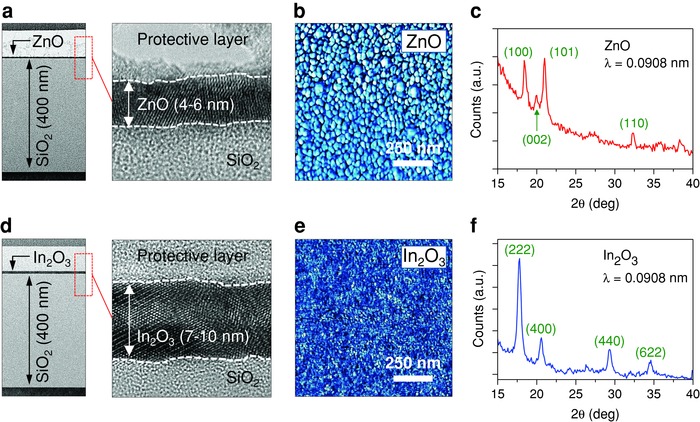
Structural analysis of low‐dimensional solution‐processed ZnO and In_2_O_3_ layers. a) High‐resolution transmission electron microscope (HRTEM) cross‐section images of the SiO_2_/ZnO interface. Left: low‐magnification TEM image confirms an ultrathin and continuous ZnO film. Right: higher‐magnification TEM image reveals the presence of polycrystalline ZnO regions with clearly visible lattice fringes. b) AFM phase images of ZnO film with individual grains clearly visible, the calculated rms ZnO surface roughness was ≈0.43 nm. c) GID measurement (X‐ray wavelength *λ* = 0.0908 nm) shows powder‐like crystallization, i.e., different crystalline orientations, coexisting in the ZnO film. d) HRTEM cross‐section images of the SiO_2_/In_2_O_3_ interfaces reveal the presence of an ultrathin and smooth In_2_O_3_ film with highly oriented crystalline domains. e) AFM image indicates the surface roughness of the In_2_O_3_ film is ≈0.20 nm. f) GID analysis (X‐ray wavelength *λ* = 0.0908 nm) shows similar powder‐like crystallization when characterizing the In_2_O_3_ layers.

We have recently demonstrated the ability to grow ultra‐thin layers of ZnO by spin casting a suitable precursor solution.^31^ Using the same aqueous precursor route, we have grown polycrystalline ZnO layers with thicknesses in the range of 3–10 nm (**Figure** **1**a) at 180 °C (see Experimental Section). As‐grown ZnO layers are found to be continuous and conformal with root mean square (rms) surface roughness of ≈0.43 nm as determined by atomic force microscopy (AFM) (Figure 1b). The polycrystalline nature of the ZnO films was also confirmed by grazing incident diffraction (GID) measurements (Figure 1c). The results suggest that ZnO layers exhibit powder‐like diffraction peaks (i.e., no preferred orientation) in agreement with the transmission electron microscope (TEM) data in Figure 1a. It is worth noting that the high‐resolution GID results reported here are the first to reveal the polycrystalline nature of these ultrathin ZnO layers as compared to early work^32^ where only the (002) peak was detected. Using previously reported methods,^33^ we have also grown ultrathin layers (5–10 nm) of In_2_O_3_ (Figure 1d) by spin casting an aqueous solution of indium nitrate [In(NO_3_)_3_] at room temperature followed by thermal annealing at ≈200 °C in air. In_2_O_3_ films are found to be continuous and conformal (Figure 1d,e), ultra‐smooth (rms ≈0.2 nm, Figure 1e) and highly polycrystalline (evidence of which are presented in Figure 1d,f) in good agreement with previously reported data.^34^ Similarly, ultrathin (2–5 nm) films of stoichiometric Ga_2_O_3_ were also grown using the same processing steps (Figure S1, Supporting Information).^35^ Unlike ZnO and In_2_O_3_, however, Ga_2_O_3_ layers appear to be largely amorphous with no signs of crystallinity as no diffraction peaks could be detected.

### 2.2 Optical Characterization of Low‐Dimensional Metal Oxide Layers

Reducing the semiconductor thickness to such extreme dimensions is also expected to impact the physical properties of the resulting oxide layers due to energy quantization phenomena.^36^, ^37^ This is expected to lead to a widening of the energy bandgap of the semiconductor as compared to its bulk value with reducing layer thickness (*L*). To investigate this effect, we performed optical absorption spectroscopy measurements in several solution‐processed ZnO and In_2_O_3_ layers of variable thickness and calculated the optical bandgap using Tauc analysis^38^, ^39^ (see Experimental Section). If we define the dimension perpendicular to the substrate surface as (z), then the energy of conduction band and valence band states available to electrons and holes, respectively, confined to an infinite quantum well (QW) can be described by^36^

(1) $$E_{n,e}=E_{xy}+\frac{n^{2}h^{2}}{8m_{e}^{*}L^{2}}$$

(2) $$E_{n,h}=E_{xy}-\frac{n^{2}h^{2}}{8m_{h}^{*}L^{2}}$$

**Figure 2:**
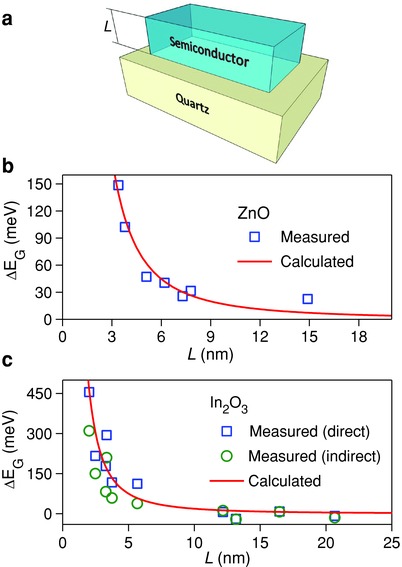
Energy quantization in solution‐processed ZnO and In_2_O_3_ layers. a) Schematic illustration of the sample configuration used for the optical absorption measurements where *L* represents the layer thickness of the spin‐coated oxide semiconductor. b) Plot of the change in optical bandgap (Δ*E*_G_) calculated via Tauc analysis versus film thickness (*L*) for several ZnO layers with respect to that of bulk ZnO (*L* > 20 nm). c) Plot of Δ*E*_G_ calculated via Tauc analysis assuming a direct (squares) and an indirect (circles) bandgap, versus film thickness for several In_2_O_3_ layers with respect to that of bulk In_2_O_3_ (*L* > 20 nm). In both plots, the solid red lines illustrate the calculated Δ*E*_G_ for an infinite quantum well using Equation (3).

Here, *E_xy_* is the energy associated with the carrier in the (unconfined) *x,y*‐plane, *n* is a positive integer, *h* is the Planck Constant, $m_{e}^{*}$ and $m_{h}^{*}$ are the effective masses of the electron and hole in the semiconductor, respectively, and *L* is the thickness of the QW in the *z*‐direction (**Figure** **2**a). As *L* is reduced the energy of the first electron state (*n* = 1), and hence the conduction band minimum (CBM), increases. Similarly the energy of the first hole state (*n* = 1), and hence the valence band maximum (VBM), decreases. As a result, the energy of the first allowed transition from the VBM to the CBM in the confined direction increases resulting in a blue‐shift in the onset of the optical absorption. The energy increase (Δ*E*_G_) as a result of *L* can then be written as^40^

(3) $$\Delta E_{G}=\frac{h^{2}}{8L^{2}}\left(\frac{1}{m_{e}^{*}}+\frac{1}{m_{h}^{*}}\right)$$

Since the bandgap of the quartz substrates used is extremely large (≈8.9 eV), the single layers of ZnO and In_2_O_3_ were modeled as infinite QW. Finite QW energies were also calculated using the known techniques,^41^ but the results were found to be negligibly different from those evaluated using the infinite QW approximation (Equation (3)). The values for the effective mass of holes ($m_{h}^{*}$) and electrons ($m_{e}^{*}$) in In_2_O_3_ employed were $m_{e}^{*}$ = 0.3 *m*_e_ and $m_{h}^{*}$ = 0.6 *m*_e_;^42^ where *m*_e_ is the rest‐mass of an electron in a vacuum. Values of effective mass for ZnO were $m_{e}^{*}$ = 0.29 *m*_e_ and $m_{h}^{*}$ = 1.2 *m*_e_.^43^, ^44^

Figure 2b,c display the measured change in the energy bandgap (Δ*E*_G_) as a function of *L* extracted using Tauc analysis (see Experimental Section). The solid lines in each plot represent the theoretical values for Δ*E*_G_ calculated using Equation (3). Good agreement between the experimental determined and the theoretically predicted Δ*E*_G_ is observed for both material systems. In the case of ZnO layers, reducing *L* leads to maximum Δ*E*_G_ values close to ≈150 meV, while in the case of In_2_O_3_ this energy difference is much larger and approaches values close to ≈450 meV. Interestingly, for In_2_O_3_ layers the extracted Δ*E*_G_ is in good agreement, within experimental error, with values calculated assuming a direct or an indirect bandgap (see Experimental Section). The noticeable differences in the measured Δ*E*_G_ values for ZnO and In_2_O_3_ layers (Figure 2b,c) are attributed to various effects the most important of which include i) the difference in the effective hole masses, ii) the differences in the conduction band energies, and iii) the different degrees of crystallinity characterising each system. On the basis of these data, we conclude that reducing the thickness of the ZnO and In_2_O_3_ layers results in a characteristic widening of the optical bandgap in good agreement with theoretical predictions for energy quantization.

The elemental compositions of the discrete metal oxide films were verified by X‐ray photoelectron spectroscopy (XPS) (Figure S1–S3) while their valence band structure was studied using ultraviolet photoelectron spectroscopy (UPS) (Figure S4, Supporting Information). The Fermi energies (*E*_F_) were estimated from Kelvin probe (KP) measurements performed in nitrogen (Figure S5, Supporting Information), yielding *E*_F_ ≈ 4.32 eV, *E*_F_ ≈ 4.65 eV, and *E*_F_ ≈ 4.90 eV for ZnO, In_2_O_3_, and Ga_2_O_3_, respectively. The optical bandgap (*E*_G_) for each discrete metal oxide layers was obtained via Tauc analysis of the absorption spectra (Figure S6, Supporting Information).

#### 2.3 Structural Characterization of Metal Oxide Heterojunctions and Quasi‐Superlattices

**Figure 3:**
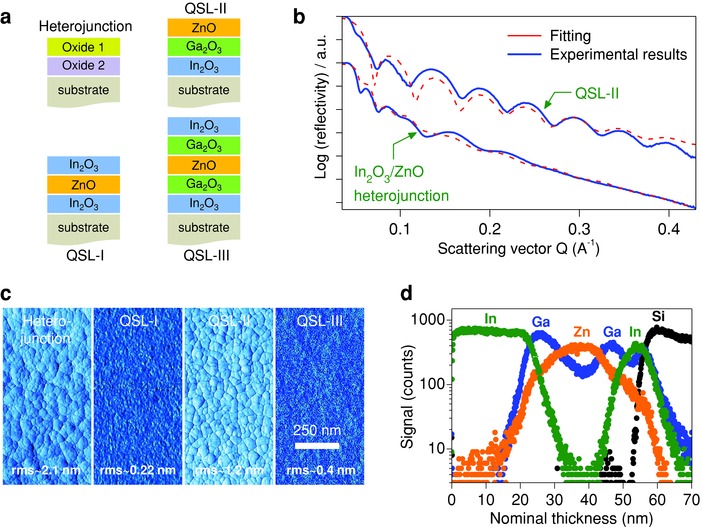
Structural analysis of metal oxide quasi‐superlattices (QSLs). a) Schematics of the different layered structures grown including heterojunctions (In_2_O_3_/ZnO, In_2_O_3_/Ga_2_O_3_, ZnO/Ga_2_O_3_) and multilayer QSLs consisting of In_2_O_3_/ZnO/In_2_O_3_ (QSL‐I), In_2_O_3_/Ga_2_O_3_/ZnO (QSL‐II), and In_2_O_3_/Ga_2_O_3_/ZnO/Ga_2_O_3_/In_2_O_3_ (QSL‐III). b) Measured (blue lines) and calculated (red‐dashed lines) XRR spectra of the In_2_O_3_/ZnO and QSL‐II layered structures. c) AFM surface phase images of the different structures namely; heterojunction, QSL‐I, QSL‐II, and QSL‐III. d) TOF‐SIMS analysis for a QSL‐III grown on Si^++^/SiO_2_ substrate. Here a thicker top layer of In_2_O_3_ (≈25 nm) was employed in order to stabilize the ion beam during measurement.

Exploring whether the sequential spin‐coating method can be applied to fabricate high‐quality oxide heterointerfaces, we grew multilayer oxide stacks based on combinations of ZnO, In_2_O_3,_ and Ga_2_O_3_. **Figure** **3**a displays schematics of the different layered structures grown including heterojunctions (In_2_O_3_/ZnO) and multilayer QSLs consisting of In_2_O_3_/ZnO/In_2_O_3_ (QSL‐I), In_2_O_3_/Ga_2_O_3_/ZnO (QSL‐II), and In_2_O_3_/Ga_2_O_3_/ZnO/Ga_2_O_3_/In_2_O_3_ (QSL‐III). Unlike conventional a‐IGZO TFTs,^45^, ^46^ here we use binary metal oxides for each layer in the stacked structures. This strategy helps to avoid complex and in some cases unwanted chemical interactions between different precursor molecules that are generally known to degrade the electrical performance of solution‐processed oxide TFTs.^4^, ^30^, ^47^

The interfacial nature of these solution‐grown metal oxide systems was investigated using X‐ray reflectometry (XRR) (Figure 3b and S7, Supporting Information). Obtained results show clear interference fringes in good agreement with theoretical simulations (Figure 3b) and in support of the existence of well‐defined binary layers with abrupt interfaces (Figure S7, Supporting Information). Table S2 (Supporting Information) summarizes the parameters used to fit (dash lines) the experimental data (solid lines) in Figure 3b and S7 (Supporting Information). Surprisingly, the XRR data show less oscillating fringes for monolayer than multilayer samples. This finding suggests that the interface roughness (i.e., substrate roughness) is significantly higher than the film's surface roughness. However, since the surface roughness of SiO_2_ is expected to be ≤5 Å, the data most likely suggest that the extracted roughness does not originate from the substrate but from the SiO_2_/In_2_O_3_ interface. The fitting parameters in Table S2 (Supporting Information) also suggest that incorporation of the Ga_2_O_3_ interlayer in‐between In_2_O_3_ and ZnO (i.e. QSL‐I) leads to a significant reduction in the interfacial roughness. This observation is in qualitative agreement with the AFM data in Figure 3c. As the AFM measurements show, the In_2_O_3_/ZnO heterojunction structure exhibits the highest rms surface roughness (≈21 Å) followed by QSL‐II (≈12 Å). In contrast, QSL‐I and QSL‐III stacks show ultra‐smooth surfaces with rms values of ≈2.2 Å and ≈4 Å, respectively. From these results, we conclude that incorporation of Ga_2_O_3_ and In_2_O_3_ helps to planarize both the buried heterointerfaces and surfaces of the QSLs.

Elemental composition profiles as a function of depth collected with time‐of‐flight secondary ion mass spectrometry (ToF‐SIMS) provide further direct evidence for the existence of sharp interfaces between the different oxide layers in QSL‐III. Figure 3d shows the Poisson corrected ion signals as a function of nominal thickness (air interface at *x* = 0 nm). Both the leading and trailing edges of the ion signal are indicative of the presence of sharp chemical interfaces between the In_2_O_3_, Ga_2_O_3,_ and ZnO. The gradual increase in Zn^+^ signal within the Ga_2_O_3_ layer and concurrent loss of Ga^+^ dynamic range is a classic example of interfacial broadening due to interfacial roughness—a feature also seen in the cross‐sectional TEM images of the individual layers shown in Figure 1a. Therefore, on the basis of the XRR (Figure 3b and S7, Supporting Information) and ToF‐SIMS (Figure 3d) data, we conclude that the solution‐grown oxide multilayers are indeed composed of ultra‐thin alternating layers separated by well‐defined interfaces. Furthermore, the existence of different ions at well‐defined depths seen in the ToF‐SIMS data (Figure 3d) indicates relatively limited intermixing.

##### 2.4 Electron Transport Measurements

**Figure 4:**
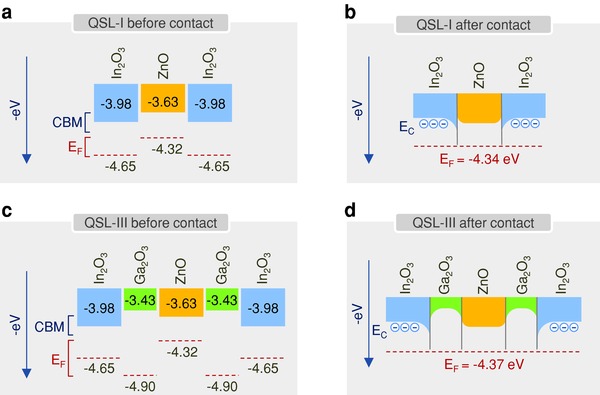
Energy levels of the metal oxide semiconductors. a) Measured energy levels of the individual oxides used in QSL‐I before contact. b) Schematic energy band diagram of QSL‐I after contact. c) Energy levels of the individual oxides used in QSL‐III before contact. d) Schematic energy band diagram of QSL‐III after contact. The energy bandgaps, Fermi energy levels, and valence band maximum (VBM) energy for each oxide material were determined using UV–vis absorption, KP and UPS measurements, respectively (see Figures S4–S6, Supporting Information).

**Figure** **4**a displays the measured energy levels of the individual oxide layers used to form the heterojunction and QSL‐I, before contact. In contrast to previously published studies in which bi‐layered metal oxide structures and transistor channels were formed using similar chemical elements,^48^, ^49^ in the present systems, the large difference in the Fermi energies (Δ*E*_F_) between the ZnO and In_2_O_3_ layers (≈300 meV) is expected to lead to electron transfer from ZnO to In_2_O_3_ upon physical contact (Figure 4b). Since the available energy levels at the CBM in the ultrathin In_2_O_3_ are quantized (Figure 2c), the transferred electrons may well be confined in a 2D potential well. On the basis of this discussion, it is not unreasonable to assume that the confined electrons will resemble the 2DEG system formed in the MgZnO/ZnO heterointerface.^19^ However, due to the polycrystalline nature of the In_2_O_3_ layer (Figure 1f), the confined electrons are not expected to behave like classic 2DEGs since macroscopic conduction in the QSL‐I is expected to be hindered by the presence of grain boundaries that are clearly visible in the high‐resolution transmission electron microscope (HRTEM) images in Figure 1.

In the case of QSL‐II and QSL‐III, insertion of the deeper Fermi energy Ga_2_O_3_ interlayer (Figure 4c) may enhance electron migration from In_2_O_3_ and ZnO and at the same time improve electron confinement due to higher interface planarity (Table S2, Supporting Information). This process is better illustrated in the energy band diagram of QSL‐III after contact shown in Figure 4d. Additionally, Ga is known to passivate interface electron trap states owing to the presence of oxygen and/or hydroxyl groups during Ga_2_O_3_ formation.^50^, ^51^, ^52^ Therefore, the combination of these beneficial attributes may yield heterointerfaces with improved electron transporting properties as compared to simple In_2_O_3_/ZnO heterointerfaces (Figure 4b). In view of these experimental findings, we argue that in In_2_O_3_/ZnO and QSL‐I structures, a confined electron system is expected to form at the vicinity of each In_2_O_3_/ZnO heterointerface due to the mismatch between the conduction and Fermi energies of the semiconductors. Similarly, in QSL‐II and QSL‐III systems, the presence of the deeper Fermi energy Ga_2_O_3_ layer is expected to planarize the heterointerface(s) as well as improve the electron confinement due to its favorable energetics and trap passivation properties.

**Figure 5:**
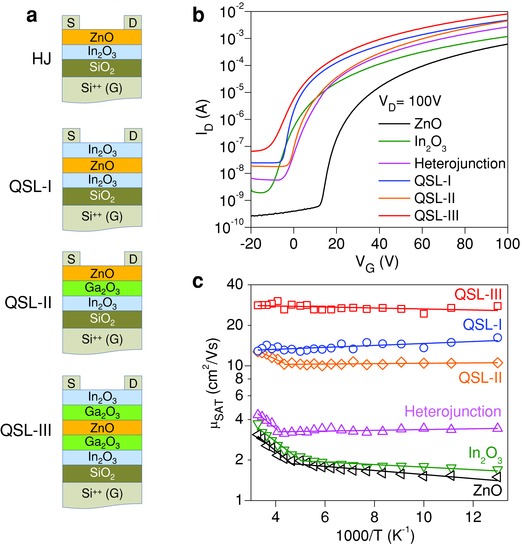
Electrical characterization of thin‐film transistor. a) Schematics of the heterojunction (HJ) and QSLs‐based metal oxide transistors developed using Si^++^ and SiO_2_ (400 nm) as the gate and the gate dielectric, respectively. b) Transfer characteristics measured from transistors with different oxide‐based channel layers including single‐layer ZnO and In_2_O_3_. c) Arrhenius plots of the temperature dependence of saturation mobility (μ_SAT_) for ZnO, In_2_O_3_, In_2_O_3_/ZnO heterojunction, QSL‐I, QSL‐II, and QSL‐III‐based transistors measured at *V*_G_ = 80 V and *V*_D_ = 100 V.

To study the nature of charge transport in these complex oxides structures, we fabricated bottom gate, top contact field effect transistors using ZnO, In_2_O_3_, Ga_2_O_3_, ZnO/In_2_O_3_, QSL‐I, QSL‐II, and QSL‐III as the channel layers (**Figure** **5**a). With the exception of Ga_2_O_3_, all samples showed n‐channel transistor behavior (Figure 5b), negligible operating hysteresis and high on/off channel current ratios (>10^5^). Representative sets of the transfer characteristics (that show both the forward and reverse sweeps) for each device are displayed in Figure S8 (Supporting Information). The room temperature electron mobilities calculated in the saturation regime (μ_SAT_) for ZnO and In_2_O_3_ ­transistors were similar and in the range of 2–4 cm^2^ V^−1^ s^−1^. Transistors based on In_2_O_3_/ZnO heterojunction channels exhibit slightly improved electron transport with maximum mobility values in the range of 3–5 cm^2^ V^−1^ s^−1^. Remarkably, we found μ_SAT_ to increase with increasing channel complexity, reaching values between 10 and 12 cm^2^ V^−1^ s^−1^ for QSL‐I and QSL‐II devices, and up to 25–30 cm^2^ V^−1^ s^−1^ for QSL‐III‐based transistors. Insertion of the Ga_2_O_3_ interlayers between In_2_O_3_ and ZnO to form QSL‐II is found to significantly improve the electron mobility of the devices while in the case of QSL‐III transistors the impact of the Ga_2_O_3_ interlayer is even greater when compared to QSL‐I. To this end the μ_SAT_ (Figure 5c) and the linear electron mobility (μ_LIN_) (Figure S9, Supporting Information) measured for optimized QSL‐III devices are among the highest reported to date for solution‐deposited metal oxide transistor channels processed at ≤200 °C.

For QSL‐I and QSL‐III transistors, the μ_SAT_ enhancement was accompanied by a threshold voltage (*V*_T_) shift to more negative gate bias, as compared to ZnO and In_2_O_3_ devices, most likely indicating the presence of a higher density of free/mobile electrons within the transistor channel (Table S3, Supporting Information).^53^ Taking into account the low‐dimensional nature of QSL and the non‐uniform field distribution across it, we argue that these additional free electrons are confined at the critical heterointerfaces (see Figure 4b,d) rather than distributed uniformly across the QSL. The increase in the off‐current in QSL‐III transistors (Figure 5b) supports this assumption since the formation of parallel channel(s) further away from the conventional dielectric/semiconductor interface will be manifested as an increase in the channel off current. Despite this, however, the devices continue to function as field‐effect transistors exhibiting excellent operating characteristics including high carrier mobility and current on/off ratios. To this end, we note that the ability to manipulate the concentration of free electrons within the confinement region by an external electric field is not unusual but on the contrary a key feature of conventional 2DEG systems.^54^, ^55^, ^56^

To examine whether the enhanced electron mobility in QSLs transistors is the result of low‐dimensional electron transport phenomena taking place at the critical In_2_O_3_/ZnO and In_2_O_3_/Ga_2_O_3_/ZnO interfaces, rather than at the conventional bottom SiO_2_/In_2_O_3_ channel interface, we carried out temperature‐dependent charge transport measurements (Figure 5c). As can be seen in single‐layer ZnO and In_2_O_3_ devices both the μ_SAT_ (Figure 5c) and μ_LIN_ (Figure S9, Supporting Information) decrease with reducing temperature down to 77 K. ZnO transistors showed consistently higher activation energies (*E*_A_) with values in the range of 28–37 meV as compared to In_2_O_3_ devices for which *E*_A_ is found to vary between 14 and 27 meV (Figures S10,S11 and Table S4, Supporting Information). ­Transistors based on In_2_O_3_/ZnO heterojunctions show similar thermally activated transport behavior but with a higher electron mobility value maintained for temperatures in the range of 77–250 K (Figure 5c, S9 and S12, Supporting Information), most likely suggesting parallel electron conduction in the upper In_2_O_3_/ZnO interface. On the contrary, electron transport in QSLs‐based devices appears to remain significantly enhanced across the entire temperature range investigated (77–300 K) with QSL‐I and QSL‐III transistors exhibiting a characteristic temperature‐independent electron mobility trend (Figure 5c, S9, S13 and S15, Supporting Information). QSL‐II devices are also found to exhibit consistently improved performance as compared to In_2_O_3_/ZnO heterojunction transistors (Figure 5c, S9 and S14, Supporting Information). The latter is attributed to the improved structural and electronic quality of the critical oxide heterointerface due to the presence of the Ga_2_O_3_ interlayer. Small but negative *E*_A_ values are calculated for the μ_LIN_ in QSL‐I and under certain biasing conditions for QSL‐III devices too (Table S4, Supporting Information). This temperature‐independent electron behavior is unique to devices based on QSL‐I and QSL‐III and is completely absent from transistors based in any other layer configuration, including QSL‐II. These findings support the idea that the nature of electron conduction in QSLs‐based devices is radically different from that in single metal oxides‐based transistors and that this difference most likely originates from the presence of free electrons confined in the vicinity of the low‐dimensional ZnO/In_2_O_3_ and In_2_O_3_/Ga_2_O_3_/ZnO interface(s) that act as parallel channels to the conventional bottom SiO_2_/In_2_O_3_ transistor channel. To this end, we emphasize that the formation of additional “conventional” parallel channels alone cannot be held solely responsible for the enhanced electron transport observed in QSL‐III transistors since such assumption will imply the somewhat unrealistic coexistence of 5–10 parallel channels in order to account for the dramatically enhanced electron mobility measured. Coexistence of several parallel conventional channels is also not expected to alter the temperature dependence of the electron transport, which should remain temperature activated and similar to that of single‐oxide transistors. It may further be argued that even if such multiple channels were to exist it would have been difficult to manipulate the free electron concentration with the gate field due to significant field attenuation occurring across each channel. Therefore, on the basis of this discussion and experimental findings, we argued that the nature of electron transport in QSLs‐based transistors is fundamentally different from the transport processes in conventional single‐oxide devices.

To better understand the charge transport characteristics of the different oxide layers, we have analyzed the interplay between two key conduction mechanisms, namely trap‐limited conduction (TLC) and percolation conduction (PC). Indeed, analysis of the gate‐field dependence of μ_LIN_ shown in Figure S9 (Supporting Information) reveals that electron transport in single metal oxide‐based transistors is dominated by a characteristic TLC mechanism (Figures S16,S17) while transport in QSL‐I/III transistors is dominated by PC—two significantly different transport processes. This difference is most likely attributed to the different electronic properties of the two active channels and particularly their Fermi energies. Specifically, in metal oxides, the high mobility states may become more accessible in systems where *E*_F_ is closer to the mobility edge.^57^, ^58^ Since the *E*_F_ in oxide QSLs is higher than that of In_2_O_3_ (Figure S5, Supporting Information), i.e., the layer in which electron transport is believed to take place, access to those highly delocalized states becomes easier hence leading to higher electron mobility. These findings further support the idea that electron conduction within the QSLs‐based devices is significantly different from that in single‐layer ZnO and In_2_O_3_‐based devices, and that it is most likely determined by the nature of the low‐dimensional oxide heterointerface(s).

##### 2.5 Depth Profiling of Electron Concentration by Capacitance‐Voltage Measurements

**Figure 6:**
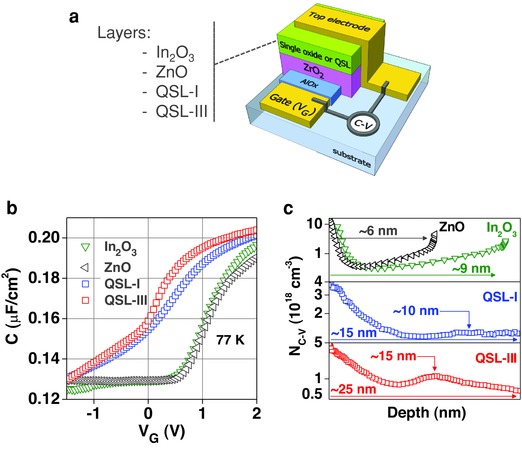
Analysis of electron confinement in metal oxide quasi‐superlattices. a) Schematic of the MIS structures used for the C–V profile analysis. b) Capacitance–voltage (C–V) measurements obtained at 77 K for ZnO, In_2_O_3_, QSL‐I‐ and QSL‐III‐based MIS devices. c) Apparent free electron (*N*_C–V_) profiles as a function of depth for MIS devices based on the different semiconducting layers calculated from the C–V data in (b).

In our effort to either prove or disprove the existence of confined electrons within the QSLs, we have attempted to determine the concentration and depth‐profile of electrons within the different oxide channels using the capacitance–voltage (C–V) profiling technique.^11^, ^59^, ^60^, ^61^ C–V measurements were performed using the metal‐insulator‐semiconductor (MIS) device structure shown in **Figure** **6**a. The hybrid AlO_X_/ZrO_2_ dielectric was chosen since QSL‐based transistors made with this system were found to yield optimum performance while its thickness is comparable to that of the semiconducting channels. In the case of semiconducting heterostructures with quantum confinement, the C–V technique enables the determination of the apparent free carrier concentration (*N*_C–V_) as well as the presence and location of the confined electrons within the heterostructure.^11^, ^59^, ^60^, ^61^ In Figure 6b, the measured C–V characteristics are presented for devices based on ZnO, In_2_O_3_, QSL‐I, and QSL‐III. MIS devices based on ZnO and In_2_O_3_ exhibit typical C–V behavior with the accumulation (*V*_G_ ≥ 2 V) and depletion regimes (*V*_G_ ≤ 0.5 V) clearly visible. QSL‐I‐ and QSL‐III‐based devices on the other hand exhibit significant differences with most notable ones: the dramatic shift of the C–V curves to more negative *V*_G_ and the appearance of differently shaped depletion regimes. To investigate the possible existence and spatial location of the confined electrons within the QSLs, we calculated the *N*_C–V_ using^59^, ^60^

(4) $$N_{C-V}=-\frac{2}{\varepsilon \varepsilon_{0}qA^{2}d\left(C^{-2}\right)/dV}$$

as a function of depth (*x*):

(5) $$x\left(V\right)=A\varepsilon \varepsilon_{0}\left(\frac{1}{C\left(V\right)}-\frac{1}{C_{\mathrm{oxide}}}\right)$$

Here, *ε* is the permittivity of the semiconductor, *ε*_0_ the dielectric constant of vacuum, and *A* is the active area of the device.

Figure 6c presents the *N*_C–V_ profiles as a function of depth, i.e., the distance from the top electrode. Unlike ZnO and In_2_O_3_ devices where *N*_C–V_ remains relatively low and uniform across the semiconductor layer, the apparent electron density within QSL‐I and QSL‐III appears consistently higher and nonuniform. For QSL‐I devices, *N*_C–V_ exhibits a clear maximum at a depth of ≈10 nm from the top electrode, which suggests the presence of confined electrons. For QSL‐III‐based devices, the electron confinement is better defined and appears at a slightly increased depth of ≈15 nm. The calculated depths for the confined electrons in both QSLs coincide with the expected position of the critical heterointerfaces as these are depicted in Figure 4b,d. Similar signatures of electron confinement are also present in C–V measurements taken at room temperature. The lack of two *N*_C–V_ maxima for QSL‐I/III devices, which would indicate the existence of a confined electron system at each critical heterointerface, is attributed to the relatively rough—compared to its thickness of ≈5 nm—nature of the central ZnO layer (Figure 1a) and the inability to resolve with high enough accuracy the two discrete electron confinement layers as these are depicted in Figure 4d. As a result, the *N*_C–V_ peak appears broader. On the basis of these findings, we conclude that a significant concentration of free electrons appears to be confined at the critical oxide heterointerfaces in accordance with the energy band diagrams of Figure 4b ,d. It should be noted that similar confinement signatures were observed in QSL‐III‐based MIS devices measured at room temperature as well as in MIS structures made on 100 nm thick SiO_2_ dielectrics.

**2.6. Quasi‐Supperlattice Metal Oxide Transistors on Rigid and Flexible Plastic Substrates**

**Figure 7:**
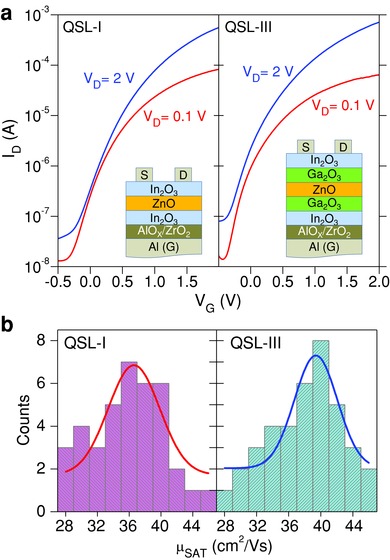
Low operating voltage transistors based on metal oxide quasi‐superlattices. a) Representative sets of transfer characteristics measured from transistors based on QSL‐I and QSL‐III channels. b) Histogram plots of the saturation mobility (μ_SAT_) calculated for a number of low‐voltage QSL‐I‐ and QSL‐III‐based transistors fabricated on the same substrates. The Gaussian fitting curves are guides to the eye.

The ability to grow ultrathin layers of oxide dielectrics (e.g., ZrO_2_) and semiconducting QSLs at low temperatures enables the creation of transistors with state‐of‐the‐art electron mobility values and low‐voltage operation on arbitrary substrates. To further demonstrate the opportunities that the QSL transistor technology has to offer, we fabricated bottom‐gate, top‐­contact transistors on glass and plastic substrates employing the AlO_X_/ZrO_2_ as the gate dielectric (see Experimental Section). The bottom‐gate‐staggered device geometry used was similar to that used for transistors made on Si/SiO_2_ with only exceptions being the gate electrode and gate dielectric materials employed. Because of the thin (≈25 nm) and high‐*k* (≈9) nature of the bilayer AlO_X_/ZrO_2_ gate dielectric (*C*_i_ ≈235 nF cm^−2^),^31^ ­as‐prepared QSL‐I/III transistors operate at significantly reduced voltages (**Figure** **7**a). QSL‐I transistors are found to exhibit consistently slightly lower mobility than QSL‐III devices with a mean value (μ_SAT(mean)_) of ≈37 cm^2^ V^−1^ s^−1^ as compared to the record value of ≈40 cm^2^ V^−1^ s^−1^ for QSL‐III devices (Figure 7b). We note that both mobility values are higher than those obtained for SiO_2_‐based transistors (Figure 5c). This ­difference is most likely attributed to improved microstructure of the semiconducting layers due to the epitaxial‐like growth on the top of the polycrystalline dielectric.^31^, ^47^, ^62^, ^63^, ^64^ The low operating voltage transistors also exhibit respectable on/off current ratios (≈10^4^) and mean subthreshold swings (SS = d*V*_G_/d[log(*I*_D_)]) of ≈275 mV dec^−1^ and ≈160 mV dec^−1^ for QSL‐I and QSL‐III devices (Figure S18, Supporting Information), respectively.

Finally, low operating voltage oxide QSL transistors were also fabricated on flexible polyethylene ­naphthalate (PEN) plastic substrates. As‐prepared devices showed reduced performance with μ_SAT(mean)_ of ≈8.5 cm^2^ V^−1^ s^−1^and ≈11 cm^2^ V^−1^ s^−1^ for QSL‐I and QSL‐III transistors, respectively (Figures S19 and S20, Supporting Information). The reduced mobility values are attributed to the lower annealing temperature (<175 °C) used in order to avoid damaging the PEN substrate during the sequential spin‐cast annealing steps used to grow the QSLs (see Experimental Section). Despite the mobility reduction, however, low‐voltage QSL‐based transistors are found to consistently outperform, in terms of electron mobility, low operating voltage transistors based on single‐layer ZnO and In_2_O_3_ channels fabricated on either glass (μ_SAT_ ≈ 3–5 cm^2^ V^−1^ s^−1^) or PEN (μ_SAT_ ≈ 1–3 cm^2^ V^−1^ s^−1^) substrates (Figures S21 and S22, Supporting Information), respectively, clearly demonstrating the advantage of the proposed oxide QSL technology over conventional single‐oxide‐layer transistors.

## 3 Conclusions

In conclusion, we have demonstrated a new concept of solution‐processed metal oxide QSL transistors. In contrast to conventional single metal oxide devices, the performance level of these QSL‐based transistors is not limited by the bulk carrier mobility of the individual semiconductor(s) involved but instead is determined by the structural and electronic properties of the oxide heterointerfaces buried within the QSL. Already, our proof of concept transistors show dramatically enhanced electron mobilities (>40 cm^2^ V^−1^ s^−1^) that far exceed values reported previously for solution‐processed metal oxide transistors fabricated at ≤200 °C^22^, ^26^, ^27^, ^28^ while they compare favorably with oxide heterojunction‐based transistors manufactured by different vacuum‐based techniques (Table S1, Supporting Information). On the basis of these results, we argue that further engineering of the band structure of the oxide QSLs—e.g., through appropriate material combinations and/or suitable chemical doping—could lead to even higher device performance and enable the design and fabrication of devices, circuits, and systems on arbitrary substrate materials via spin‐casting or other large‐area compatible deposition methods such as spray pyrolysis, printing as well as numerous vacuum‐based techniques. The unique combination of low‐cost, low‐temperature processing with the exceptionally high electron mobility achieved can potentially fulfill the ever increasing demand for high‐performance thin‐film transistor technologies across a wide range of applications spanning from next‐generation ultra‐high‐definition optical ­displays to future generations of transparent electronics.

## 4 Experimental Section

*Oxide Precursor Preparation and Processing*: Zn amine complex solutions were prepared by dissolving ZnO hydrate (ZnO·xH_2_O, 97%; Sigma–Aldrich) in ammonium hydroxide (50% v/v; Alfa Aesar) at 10 mg mL^−1^. As‐prepared solutions were then stirred rigorously at room temperature for 2 h. This process yielded a clear transparent Zn ammine complex‐based solution. For the growth of In_2_O_3_ layers, the precursor solution was prepared by dissolving anhydrous indium nitrate (In(NO_3_)_3_, 99.99%; Indium Corporation) in deionized (DI) water at a concentration of 30 mg mL^−1^. The solution was subjected to rigorous stirring at room temperature for 60 min before use. For solution‐processable gallium oxide (Ga_2_O_3_), the precursor solution was prepared by dissolving gallium nitrate hydrate (Ga(NO_3_)_3_·xH2O, 99%; Sigma–Aldrich) in DI water at a concentration of 10 mg mL^−1^. The solution was also stirred at room temperature for 1 h before use. For ZrO_2_ deposition, the precursor solution was synthesized by dissolving Zr(IV) acetylacetonate (Zr(C_5_H_7_O_2_)_4_) (98%; Sigma–Aldrich) in *N,N*‐dimethylformamide (DMF, C_3_H_7_NO) (Sigma–Aldrich) at a concentration of 0.15 m in inert gas atmosphere with the addition of an equal molar concentration of ethanolamine (MEA, C_2_H_7_NO) (≥99%; Sigma–Aldrich). The solution was then subjected to rigorous stirring at 70–80 °C for 1 h before use.

*Substrate Preparation*: Heavily doped silicon (Si^++^) wafers acting as the common gate electrode and a thermally grown SiO_2_ layers (400 nm) as the gate dielectric were used as the transistor substrates. Prior to spin‐casting the semiconductor layer, the substrates were cleaned by sonication in a solvent bath lasting for ≈10 min. The four steps were: 1) sonication in DI water with 2 mL Decon 90, 2) sonication in DI water, 3) sonication in acetone, 4) followed by sonication in isopropanol. The residual solvent was then dried by blowing dry nitrogen over the surface of the substrates. Finally, the substrates were exposed to UV ozone for 10 min to remove residual hydrocarbons from the SiO_2_ surface. All other types of device and sample substrates used in this work were cleaned using the same process protocol, except for plastic films (i.e., PEN, Teijin Dupont Films) for which the sonication bath step was performed using purified DI water in order to remove dust particles and other impurities.

*Metal Oxide Layers Deposition and Transistor Fabrication*: For the In_2_O_3_ and ZnO TFTs, the semiconductor thin‐film deposition was carried out by spin‐casting the precursor solutions onto the Si^++^/SiO_2_ substrates at 4000 rpm for 30 s in ambient air, followed by a post‐deposition thermal‐annealing process for 30 min at 180–200 °C in ambient air. The overall layer thickness was controlled by the number of deposition steps performed. For single‐layer ZnO and In_2_O_3_ transistors, semiconductors were grown using a two deposition step process in order to increase the overall thickness of the channel layer. Fabrication of the In_2_O_3_ and ZnO transistors was completed with the thermal evaporation of 40 nm thickness Au and Al top source and drain (S–D) electrodes through a shadow mask in high vacuum (≈10^−6^ mbar), respectively. Fabrication of heterojunction and QSL‐based transistors was performed using identical spin‐casting and thermal‐annealing conditions to those used for the fabrication of single‐layer ZnO and In_2_O_3_ transistors with the exception that each layer growth was performed using a single‐step deposition in order to maintain a similar channel layer thickness with that of single‐oxide devices. Similarly, deposition of Ga_2_O_3_ layers was carried out using a single deposition step. Top S–D electrodes in QSLs transistors were formed by thermal evaporation of 40 nm thick Au through a shadow mask in high vacuum. For transistors prepared on Si/SiO_2_ wafers, the channel width (*W*_ch_) and length (*L*_ch_) were 1000 and 50 μm, respectively, while for the low operating voltage transistors the channel dimensions of *W*_ch_ = 1000 μm and *L*_ch_ = 30 μm, were employed. For each transistor configuration, the semiconducting layer(s) was patterned by wiping the as‐deposited wet precursor film prior to thermal annealing/conversion. The latter process step together with the use of large *W*_ch_/*L*_ch_ ratios (≥20) ensured minimum contribution of fringing currents and elimination of associated errors in electron mobility calculations.

*Absorption Spectroscopy of Metal Oxide Films*: Several ZnO and In_2_O_3_ films were spin‐cast onto quartz substrates from the precursor solutions in air. As‐spun layers were then annealed at 200 °C for 30 min in air. UV–vis absorption measurements were carried out with a Shimadzu UV‐2600 UV–vis spectrophotometer. Transmittance and reflectance measurements were carried out for each sample. The transmittance corrected for reflectance was derived from the raw transmittance + raw reflectance.

Tauc analysis^38^, ^39^ was used to approximate the bandgap of each film. The technique entails plotting (*αhν*)*^X^* against the incident photon energy (*hν*), then extrapolating the linear part of the plot to (*αhν*)*^X^* = 0. Here, *α* is the optical absorbance of the material and *X* is an exponent that depends on the nature of the semiconductor bandgap, i.e., direct or indirect. For direct bandgap semiconductors *X* = 2 is used, whilst for indirect bandgap semiconductors *X* = 1/2 is used. Since the nature of the bandgap in In_2_O_3_ is still under debate,^65^, ^66^, ^67^ we have here used both approaches. ZnO is known to be a direct bandgap semiconductor^68^ thus the value of *X* = 2 was employed. By assuming that the optical properties of layers with thickness >20 nm are representative of the bulk semiconductors, the increase in bandgap (Δ*E*_G_) relative to the bulk was evaluated for each semiconducting layer.

*Processing of High‐k Dielectrics for Metal‐Insulator‐Semiconductor Capacitors and Low‐Operating Voltage Transistors*: The metal‐insulator‐semiconductor capacitors and low‐voltage transistors were fabricated using a combination of metal oxides as the dielectric layers. Devices were fabricated on glass as well as plastic substrates. Following substrate cleaning, 40 nm thick Al gate electrodes were deposited by thermal evaporation through a shadow mask. The native aluminum oxide was grown on the surface of the Al gate electrodes using a low‐pressure mercury UV lamp, which emits at wavelengths of 253.7 nm (97% of overall power) and of 184.9 nm (3% of overall power) at total output power of approximately 5 mW cm^−2^ (at a distance of 1 cm). The entire UV illumination was taken in ambient air for 10–12 h. Following, the ZrO_2_ film was grown by spin‐coating the precursor solution at 3000 rpm for 60 s in nitrogen followed by curing the samples using a metal halide lamp of 250 mW cm^−2^, equipped with a UVA spectrum filter, for 90 min in ambient air.

*Grazing Incident Diffraction and X‐Ray Reflectivity Measurements*: Grazing incident diffraction and X‐ray reflectivity were carried out on beamline G2 in Cornell High Energy Synchrotron Source (CHESS) at Cornell University (USA). The samples were aligned on a Kappa diffractometer with the X‐ray energy of 13.65 keV (*λ* = 0.0908 nm) through a Be single‐crystal monochromator. The data were collected using a 640‐element 1D diode‐array detector, with a set of 0.1° Soller slits mounted on the detector arm to provide an in‐plane resolution of 0.16°. The grazing incident angle was fixed at 0.1° in GID. The XRR results were simulated by Parratt32 software program developed at HMI in Berlin (Germany).

*Transistor Characterization*: Device electrical characterization was carried out under high vacuum (≈10^−5^ mbar) at temperature ranging from 77 to 305 K using a cryogenic probe station (Janis ST‐500). Electrical measurements were carried out with a Keithley 4200 semiconductor parameter analyzer. Electron mobility was extracted from the transfer curves in the linear/saturation regime using the gradual channel approximation:

(6) $$\mu_{\mathrm{LIN}}=\frac{L_{\mathrm{ch}}}{C_{i}W_{\mathrm{ch}}}\frac{\partial I_{D}}{\partial V_{G}}\frac{1}{V_{D}}$$

(7) $$\mu_{\mathrm{SAT}}=\frac{L_{\mathrm{ch}}}{C_{i}W_{\mathrm{ch}}}\frac{\partial^{2}I_{D}}{\partial V_{G}^{2}}$$

where, *C*_i_ is the geometrical capacitance of the SiO_2_ dielectric layer, and *L*_ch_ and *W*_ch_ are the length and width of the transistor channel, respectively.

## Supporting information

As a service to our authors and readers, this journal provides supporting information supplied by the authors. Such materials are peer reviewed and may be re‐organized for online delivery, but are not copy‐edited or typeset. Technical support issues arising from supporting information (other than missing files) should be addressed to the authors.

Supplementary
