# Supplementary material for: High Electron Mobility Thin‐Film Transistors Based on Solution‐Processed Semiconducting Metal Oxide Heterojunctions and Quasi‐Superlattices
Source: Adv Sci (Weinh). 2015 May 26;2(7):1500058. doi: 10.1002/advs.201500058 (PMC5016782; doi:10.1002/advs.201500058)
Supplement: Supplementary file 1 — Supplementary [file ADVS-2-0a-s001.pdf]

Copyright WILEY-VCH Verlag GmbH & Co. KGaA, 69469 Weinheim, Germany, 2015.

ADVANCED SCIENCE
Open Access

Supporting Information

for *Adv. Sci.*, DOI: 10.1002/advs.201500058

High Electron Mobility Thin-Film Transistors Based on Solution-Processed Semiconducting Metal Oxide Heterojunctions and Quasi-Superlattices

*Yen-Hung Lin, Hendrik Faber, John G. Labram, Emmanuel Stratakis, Labrini Sygellou, Emmanuel Kymakis, Nikolaos A. Hastas, Ruipeng Li, Kui Zhao, Aram Amassian, Neil D. Treat, Martyn McLachlan, and Thomas D. Anthopoulos**

Supporting Information for

High electron mobility transistors based on solution-processed semiconducting metal oxide quasi-superlattices

Yen-Hung Lin[1,2], Hendrik Faber[1], John G. Labram[1], Emmanuel Stratakis[3,4], Labrini Sygellou[5], Emmanuel Kymakis[6], Nikolaos A. Hastas[7], Ruipeng Li[8], Kui Zhao[9], Aram Amassian[9], Neal Treat[10], Martyn McLachlan[10] and Thomas D. Anthopoulos[1,*]

[1]Department of Physics and Centre for Plastic Electronics, Blackett Laboratory, Imperial College London, London SW7 2AZ, United Kingdom
[2]Dutch Polymer Institute (DPI), P.O. Box 902, 5600 AX Eindhoven, The Netherlands
[3]Institute of Electronic Structure and Laser (IESL), Foundation for Research and Technology-Hellas (FORTH), Heraklion, 71003, Greece
[4]Materials Science & Technology Department, University of Crete, Heraklion 71003, Greece
[5]Institute of Chemical Engineering and High Temperature Processes (ICEHT), Foundation of Research and Technology Hellas (FORTH), Stadiou Strasse Platani, P.O. Box 1414, Patras GR-265 04, Greece
[6]Center of Materials Technology and Photonics & Electrical Engineering Department, Technological Educational Institute (TEI) of Crete, Heraklion, 71004, Greece
[7]Aristotle University of Thessaloniki, Physics Department, Thessaloniki 54124, Greece
[8]Cornell High Energy Synchrotron Source, Wilson Laboratory, Cornell University, Ithaca, New York 14853, United States
[9]Materials Science and Engineering, Division of Physical Sciences and Engineering, King Abdullah University of Science and Technology, Thuwal 23955-6900, Saudi Arabia
[10]Department of Materials and Centre for Plastic Electronics, Imperial College London, London SW7 2AZ, United Kingdom

*Correspondence to: E-mail: thomas.anthopoulos@imperial.ac.uk

Thin-film Characterization Methods

S1.1. Kelvin probe measurement

Fermi levels of all metal-oxide layers were taken using a KP Technology scanning Kelvin probe system SKP5050 in nitrogen environment at room temperature.

S1.2. UV-Vis-NIR spectroscopy

UV-Vis-NIR spectra of metal-oxide films were recorded with a Shimadzu UV-2600 spectrophotometer equipped with an ISR-2600Plus integrating sphere.

S1.3. Atomic force microscopy (AFM) measurements

Atomic force micrographs of the films were taken in the tapping mode using an Agilent 5500 AFM in air.

S1.4. Time-of-flight secondary ion mass spectrometry (TOF-SIMS) measurements

An IONTOF SIMS[5] at 10^{-8} torr was used to measure depth profiles with a 2kV Cs^+ (~75 nA) sputter beam rastered across a 400 × 400 μm^2. Positive ions were collected from the central 100 × 100 μm^2 of the sputter crater using a Bi_3^+ (~1 pA) beam with charge compensation.

S1.5. X-ray photoelectron spectroscopy (XPS) measurements

The surface analysis studies were performed in a UHV chamber (<10^{-9} mbar) equipped with a SPECS LHS-10 hemispherical electron analyzer. The XPS measurements were carried out at room temperature using non-monochromatic aluminium Kα X-ray radiation under conditions optimized for maximum signal: constant ΔE mode with pass energy of 36 eV giving a full width at half maximum (FWHM) of 0.9 eV for the Au 4f7/2 peak. The analyzed area was an ellipsoid with dimensions 2.5 × 4.5 mm^2. The XPS core level spectra were analyzed using a fitting routine, which allows the decomposition of each spectrum into individual mixed Gaussian-Lorentzian components after a Shirley background subtraction.

S1.6. Ultraviolet photoelectron spectroscopy (UPS) measurements

The ultraviolet photoelectron spectroscopy (UPS) spectra were obtained using HeI irradiation with hν = 21.23 eV produced by a UV source (model UVS 10/35). During UPS

measurements the analyzer was working at the Constant Retarding Ratio (CRR) mode, with CRR = 10. The work function (Φ) was determined from the UPS spectra by subtracting their width (i.e. the energy difference between the analyzer Fermi level and the high binding energy cutoff), from the HeI excitation energy. For these measurements a bias of -12.29 V was applied to the sample in order to avoid interference of the spectrometer threshold in the UPS spectra.

S1.7. Electron microscopy measurements

A transmission electron microscope operating at an accelerating voltage of 300 kV (Titan 80-300 Super Twin, FEI Company) was used to acquire cross-section micrographs. Charged couple device (CCD) camera (Model: US4000, Gatan Inc.) was used to record HR-TEM images. Samples were prepared on a focused ion beam (FIB; Helios 400s, FEI) equipped with a nanomanipulator (Omniprobe, AutoProbe300) with lift-out method. Electron beam assisted carbon and platinum deposition was performed on the sample surface to protect the thin film surface against the ion beam bombardment during ion beam milling. Ga ion beam (30 kV, 9 nA) was first used to cut the sample from the bulk (30 kV, 9 nA), after which it was attached to a Cu grid using a lift-out method. The sample was subsequently thinned down to ca. 50 nm thickness (30 kV, 93 pA) and cleaned (2 kV, 28 pA) to get rid of areas of the sample damaged during the thinning process.

Figures and Tables

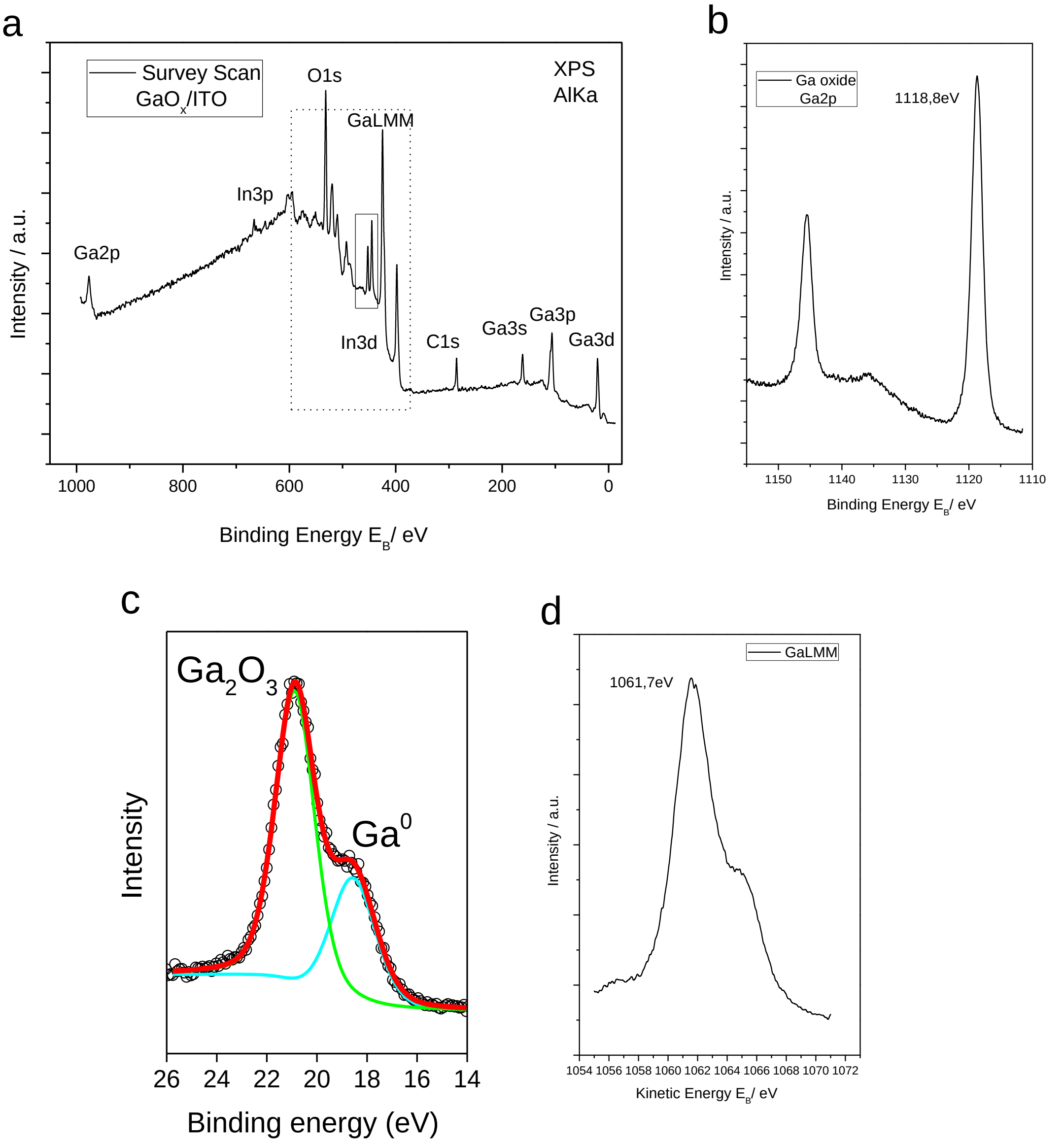

Figure S1. (a) XPS survey spectra of a Ga_2O_3 film deposited on a glass/ITO substrate. (b) and (c) shows the XPS spectra of Ga 2p, Ga 3d core level peaks respectively, and (d) the XAE spectrum of Ga LMM Auger transition. The binding energy of Ga $2p_{3/2}$ is 1118.8 eV assigned to Ga_2O_3 whereas the Ga 3d peak analyzed into two components assigned to Ga^0(18.5 eV) and Ga_2O_3 (20.9 eV).[1-3] The inelastic mean free path, λ, of electrons with kinetic energy 367.8eV for Ga $2p_{3/2}$, $\lambda_{Ga2p3/2}$ ~ 0.9nm, is three times less than that of electrons with kinetic energy 1066.7eV for Ga 3d, λ_{Ga3d} ~ 2.66nm through an inorganic matrix. Furthermore, the Ga 2p peak can be detected only under the presence of the Ga_2O_3 component. Since 95% of the measured information obtained by XPS comes from within three attenuation lengths from the sample surface, one can, therefore, conclude that the Ga_2O_3 covers the Ga film. Using the intensity ratio of the photoelectron peaks components of Ga3d and the appropriate equations and parameters we calculate that the thickness of Ga_2O_3 is 2.5 nm (± 0.1 nm) and the thickness of the underlying Ga layer is 0.5 nm (± 0.1 nm).

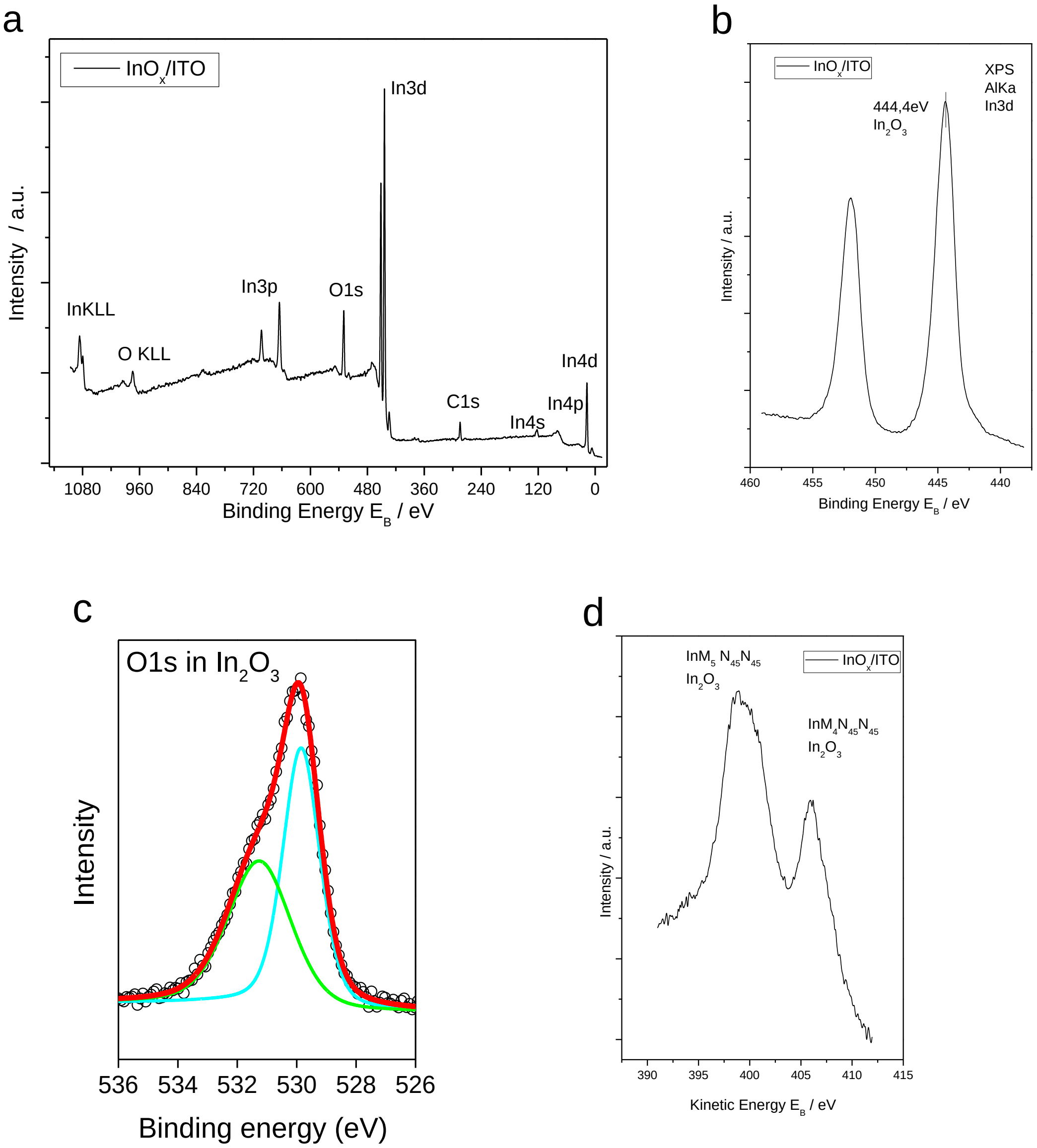

Figure S2. (a) XPS survey spectra of an In_2O_3 film deposited on a glass/ITO substrate. (b) and (c) shows the detailed XP spectra of In 3d and O1s core level peaks respectively and (d) the XAE spectrum of In MNN Auger transition. The modified Auger parameter is $\alpha' = 850.9$ eV and the In $3d_{5/2}$ binding energy is at 444.4 eV both assigned to In_2O_3.[4-6] The O1s peak in In_2O_3 generally can be deconvoluted into three different components — ~530 eV: O in oxide lattices without vacancies, ~532.0 eV: O in oxide lattices with vacancies and ~533 eV: O in hydroxide-related species.[7] From Figure S2(c) the obtained O1s peak consists of two main energies at ~530 eV and ~532 eV. Such a result reveals a high-quality In_2O_3 layer can be achieved with this aqueous-based precursor system since only a minimum (barely observable) hydroxide-related content could be found in the final film stage.[8]

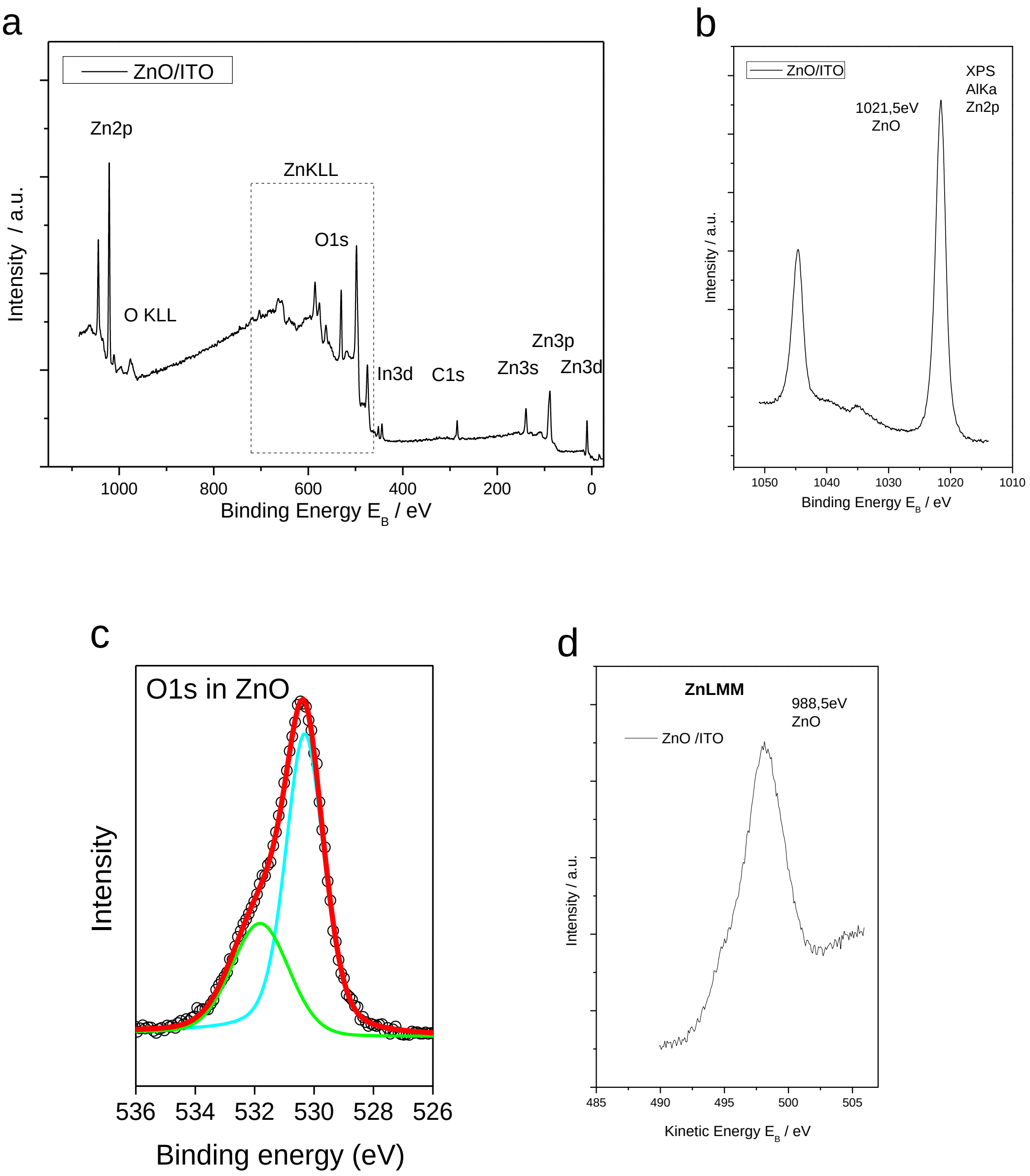

Figure S3. (a) XPS survey spectra of a ZnO film deposited onto a glass/ITO substrate. The XP Specta of Zn 2p and O1s core level peaks and the XAE Spectrum of Zn LMM Auger transition are respectively shown in (b), (c) and (d). The obtained modified Auger parameter is α' = 2010.0 (± 0.1) eV and the Zn $2p_{3/2}$ is found at 1021.7 eV binding energy both assigned to ZnO.[9-10] The O1s envelope obtained here can be deconvoluted into two main peaks that are located below 530 eV (O^{2-} in ZnO wurtzite structures) and over 532 eV (aqueous adsorbates on the surface of ZnO or Zn-hydroxide species).[11-12] The observation of hydroxide-related groups can be attributed to the re-adsorption of ambient moisture on the reactive ZnO surface and/or the existence of unconverted precursor impurities.

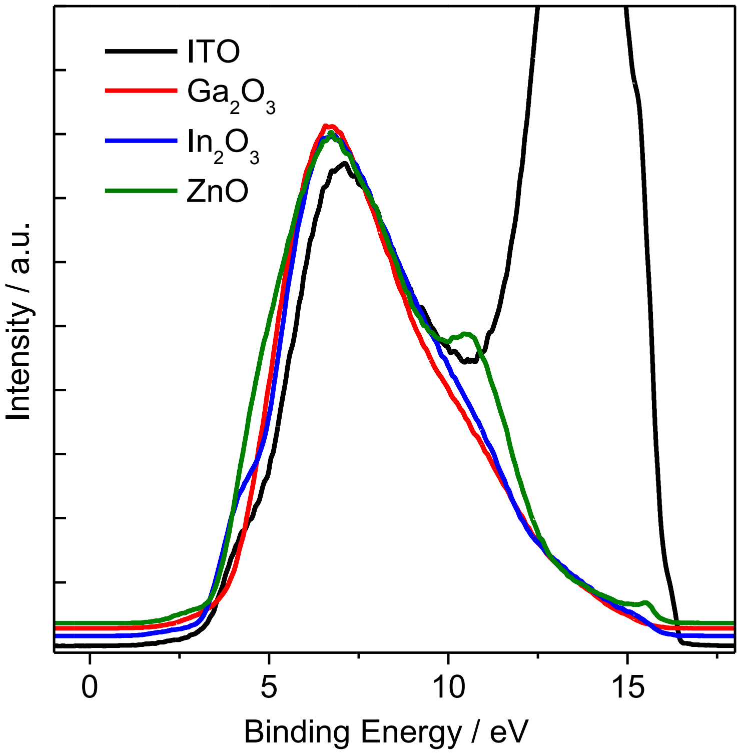

Figure S4. UPS spectra used to determine the valence band edge from the vacuum level. Extracted values were ~6.99 eV, ~7.85 eV and ~8.38 eV for ZnO, In_2O_3 and Ga_2O_3, respectively. The work function of the reference ITO electrode was ~4.73 eV. To be noted that the obtained ionized energies here have good agreement with previously reported data in the literature works.[13-14]

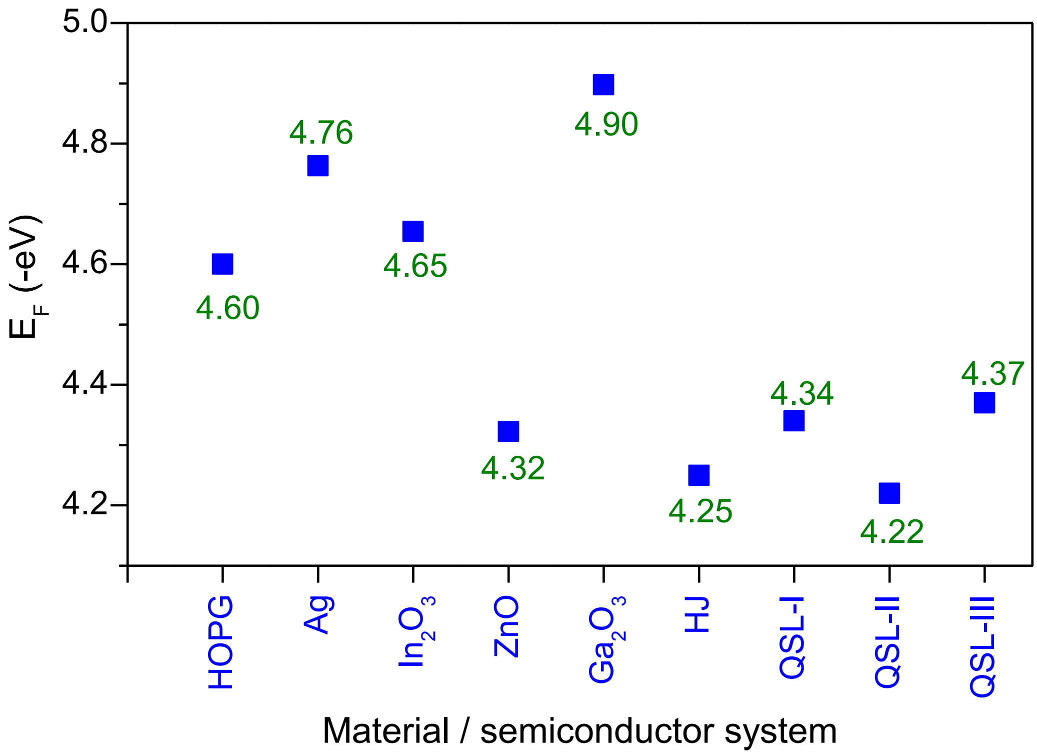

Figure S5. Kelvin Probe (KP) analysis of the Fermi energy levels of highly-ordered pyrolytic graphite (HOPG), silver (Ag), In_2O_3, ZnO, Ga_2O_3, HJ, QSL-I, QSL-II, and QSL-III.

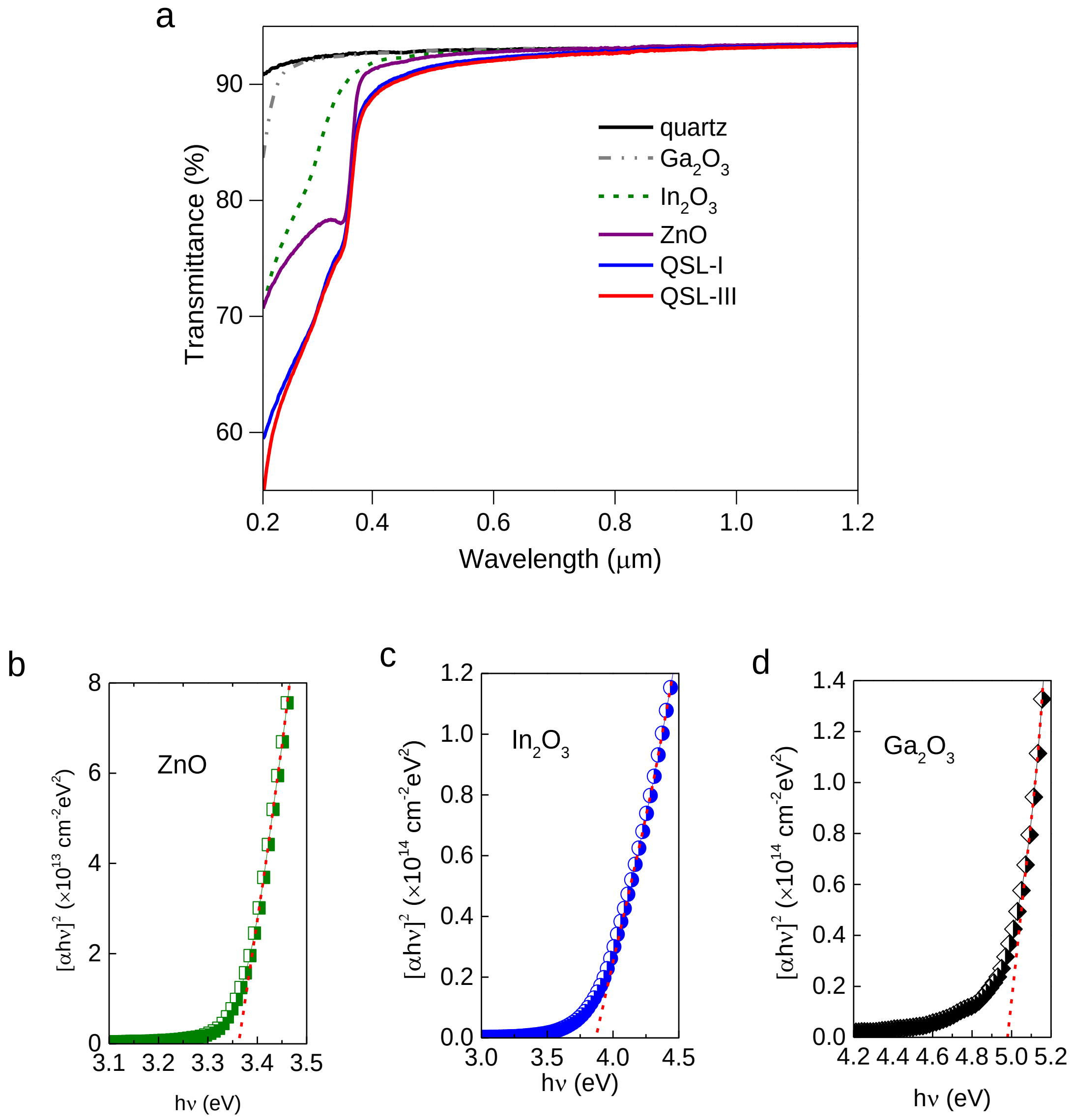

Figure S6. (a) UV-Vis-NIR transmission spectra of the blank quartz substrate, Ga_2O_3, In_2O_3, ZnO, QSL-I and QSL-III structures. The existence of discrete oxide layers in the case of QSLs, are found in the optical absorption measurements from which a superposition of the absorption spectra of the individual oxides is observed. Tauc analysis of the absorption spectra of QSL-I and QSL-III yields a similar band gap to ZnO (~3.35 eV), i.e. the smallest bandgap material in the stack.[15] Therefore we conclude that formation of well-defined hetero-junctions and QSLs with good quality hetero-interfaces is possible via sequential spin casting of the different oxides. This result is in agreement with the XRR and SIMS measurements (Figure 2b–c and Figure S7). Tauc plots of three metal oxide films: (b) ZnO, (c) In_2O_3, and (d) Ga_2O_3. The extracted optical bandgaps are 3.36 eV, 3.87 eV, and 4.95 eV for ZnO, In_2O_3, and Ga_2O_3, respectively. These results are in good agreement with the data reported in the previous works.[14, 16-17]

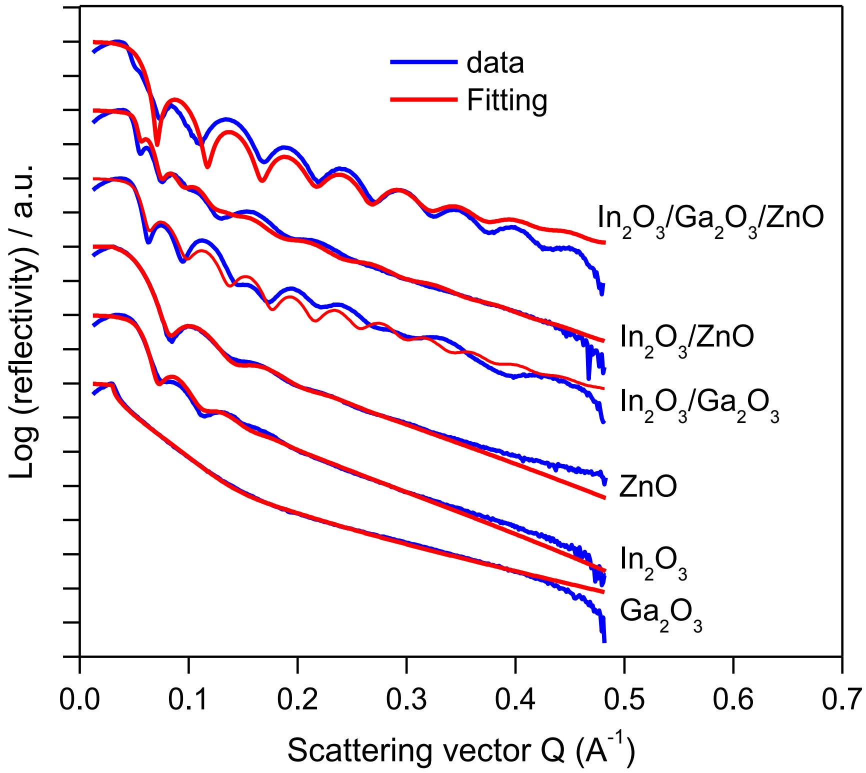

Figure S7. X-ray reflectivity (XRR) measurements obtained from single, bilayer and multilayer oxide structures. Measured and calculated spectra are shown in light-blue and red lines, respectively. For single-layer oxide films the surface roughness was calculated yielding ~3.0 Å, ~4.8 Å and ~4.5 Å for Ga_2O_3, In_2O_3 and ZnO, respectively. The interface roughness for the bilayers In_2O_3/Ga_2O_3, In_2O_3/ZnO and In_2O_3/Ga_2O_3/ZnO were also calculated yielding ~6.5 Å, ~6.2 Å and ~2.5-5.3 Å, respectively. The surface roughness values of the same oxide bilayer structures were also calculated yielding: ~3.3 Å (In_2O_3/Ga_2O_3), ~4.1 Å (In_2O_3/ZnO) and ~2.4 Å (In_2O_3/Ga_2O_3/ZnO).

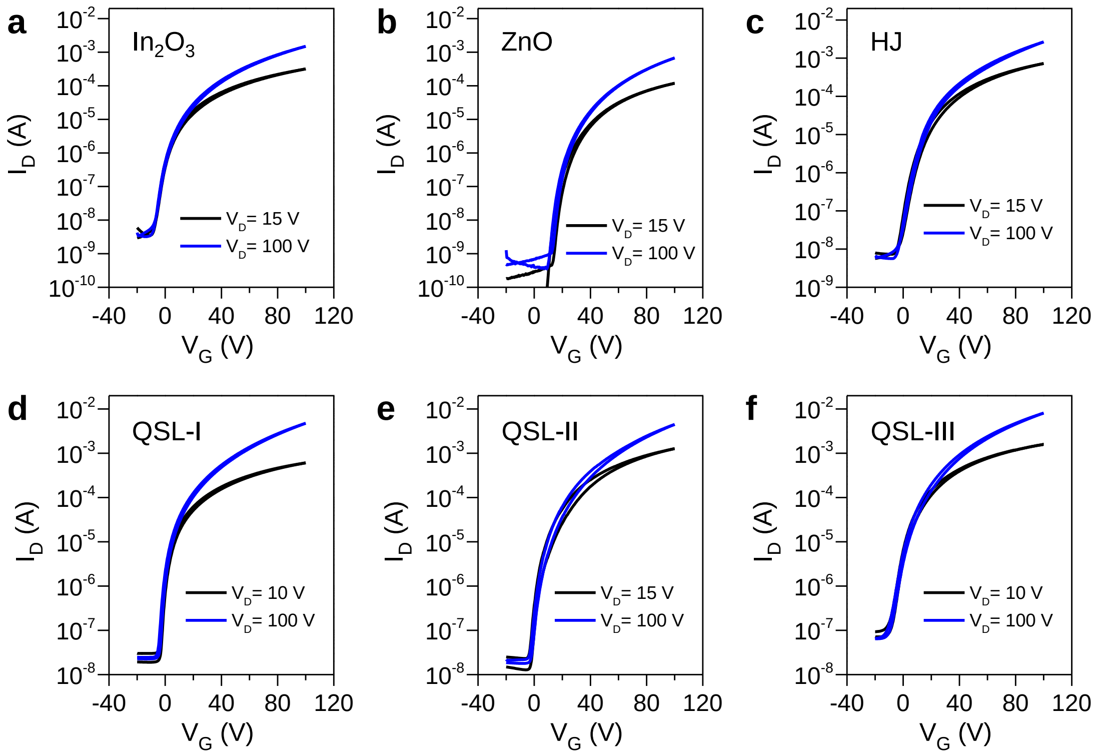

Figure S8. Representative sets of transfer characteristics measured from (a) In_2O_3, (b) ZnO, (c) heterojunction (HJ), (d) QSL-I, (e) QSL-II and (f) QSL-III transistors. All types of transistors exhibit minor operating hysteresis and excellent stability when stored under inert atmosphere (i.e. dry nitrogen).

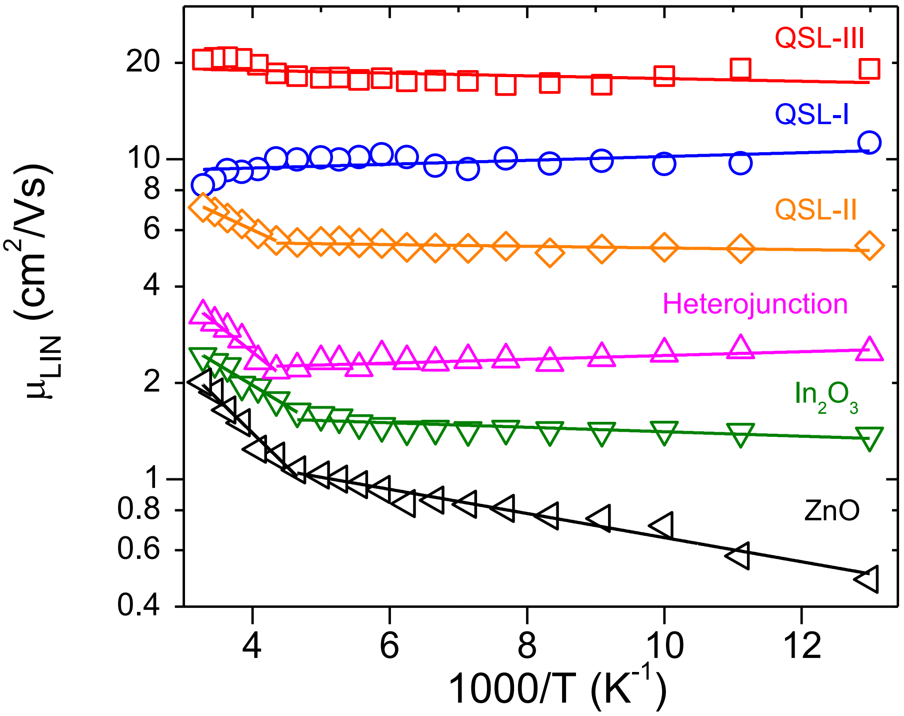

Figure S9. Arrhenius plots of the temperature dependence of the linear field-effect mobility (μ_{LIN}) measured for ZnO, In_2O_3, heterojunction, QSL-I, QSL-II and QSL-III transistors at V_G = 80 V and V_D = 15 V.

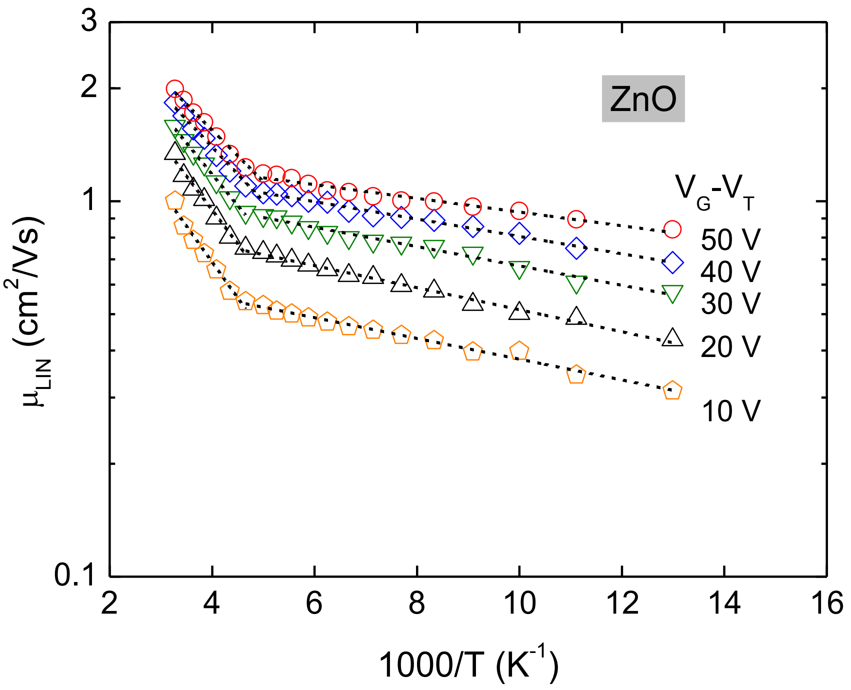

Figure S10. Arrhenius plots of the linear field-effect mobility (μ_{LIN}) calculated at different gate voltages (V_G-V_T) for a representative ZnO transistor. Two different charge transport regimes can be observed, one at T ≥ 200 K and one at T ≤ 200 K, each characterized by a different activation energy (E_A). The characteristic transition temperature of ~200 K separating the two transport regimes remains constant and independent of the gate bias (V_G). A summary of the calculated E_A for the ZnO device at T ≥ 200 K are summarized in Table S4.

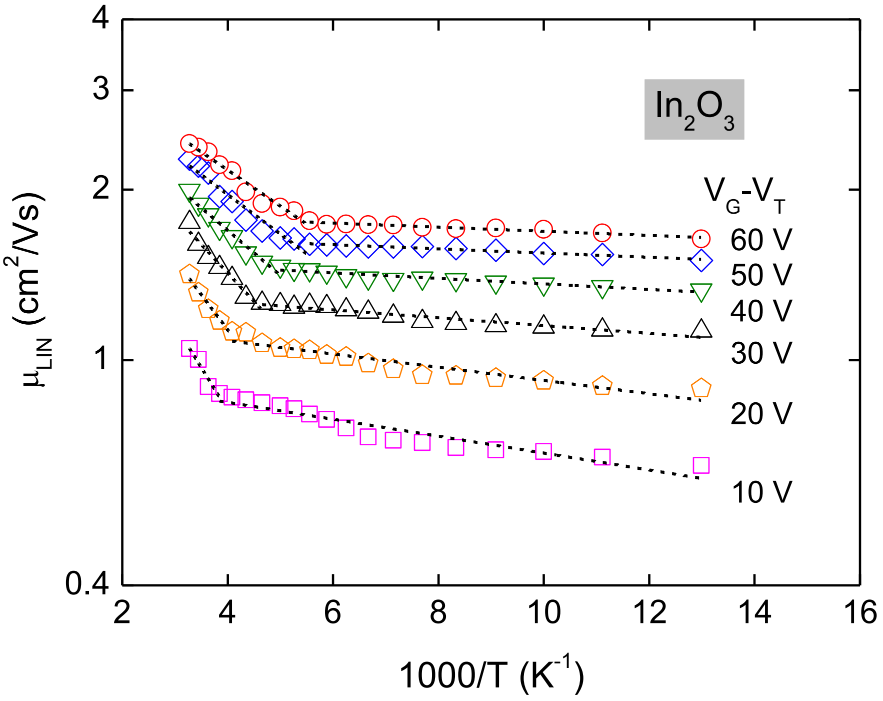

Figure S11. Arrhenius plots of the linear field-effect mobility (μ_{LIN}) calculated at different gate voltages (V_G-V_T) for a representative In_2O_3 transistor. Two discrete operating regimes, one at higher and one at lower temperatures, are observed. Unlike ZnO devices, however, in the case of In_2O_3 transistors when the V_G-V_T increases from 10 V to 60 V, the transition temperature - i.e. the temperature separating the two transport regimes - decreases from 245 K to 190 K. A summary of the high temperature range E_A calculated for the In_2O_3 transistor is given in Table S4. From these results we conclude that for ZnO and In_2O_3 the thermal energy (*k*T) for temperatures above the characteristics transition temperature is comparable to the energy range of the gap states and large enough to induce thermally activated free electrons in the channel.[18]

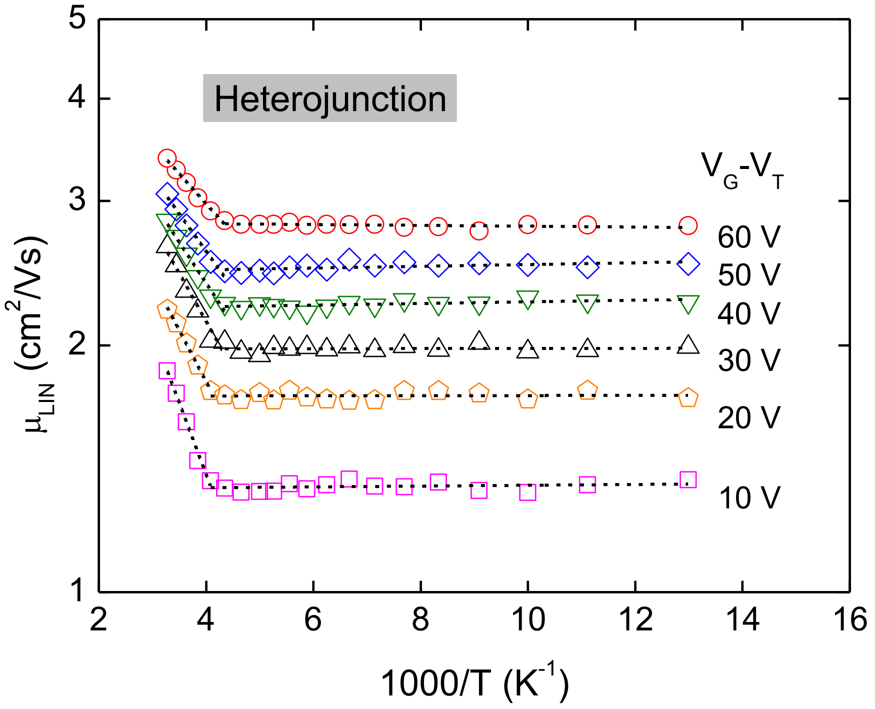

Figure S12. Arrhenius plots of the linear field-effect mobility (μ_{LIN}) calculated at different gate voltages (V_G-V_T) for an In_2O_3/ZnO transistor. After an initial drop, the electron mobility remains constant for a wide temperature range (77-250 K). For T > 250 K a thermally activated electron transport process (E_A ~19.8 meV) is observed. A summary of the calculated E_A in the high temperature range is given in Table S4.

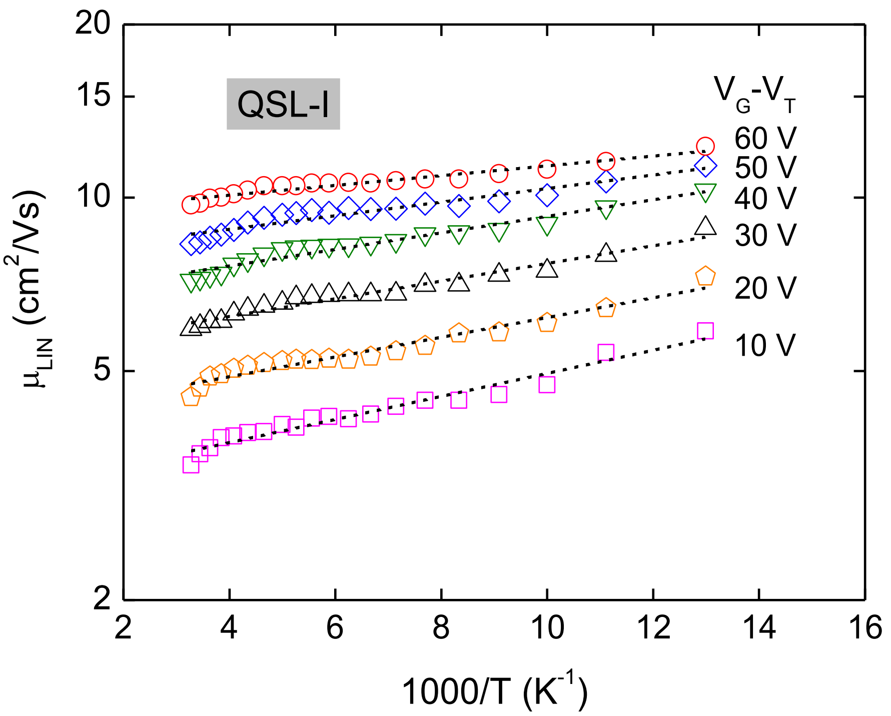

Figure S13. Arrhenius plots of the linear electron field-effect mobility (μ_{LIN}) calculated at different gate voltages (V_G-V_T) for a QSL-I transistor. Unlike ZnO, In_2O_3, and In_2O_3/ZnO transistors, the electron mobility increases with decreasing temperature for all gate biases investigated. This characteristic enhancement in electron mobility at lower temperatures is attributed to the formation of quasi 2DEG carriers at the vicinity of the critical heterointerfaces in agreement with previous reports.[19]

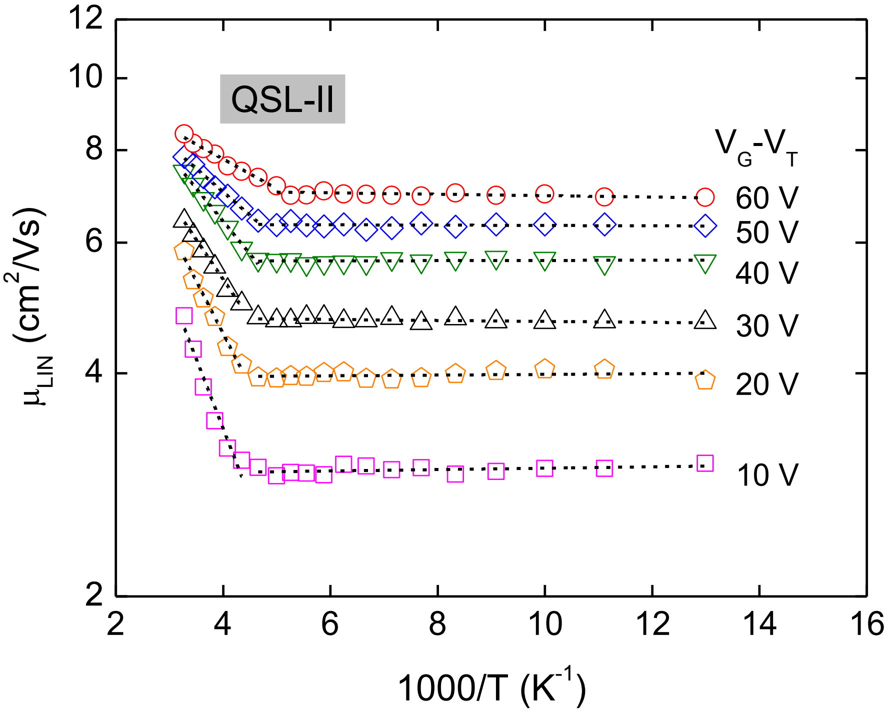

Figure S14. Arrhenius plots of the linear field-effect mobility (μ_{LIN}) calculated at different gate voltages (V_G-V_T) for QSL-II transistors. A thermally activated electron transport is observed at temperatures in the range 220-230 K with an E_A value of ~19-20 meV. Importantly, the device show higher electron mobility with a reduced temperature dependence. This is most likely attributed to the conduction band bending at/near the hetero-interface(s) where free electrons can migrate to and fill the conduction-band tail states as well as the quasi "2DEG" states owing to the internal electric field built up.[19] This hypothesis can be further studied by analyzing the percolation threshold voltage (V_P) of each device (see Figure S17). The E_A values calculated at 220-230 K for the QSL-II transistor are summarized in Table S4.

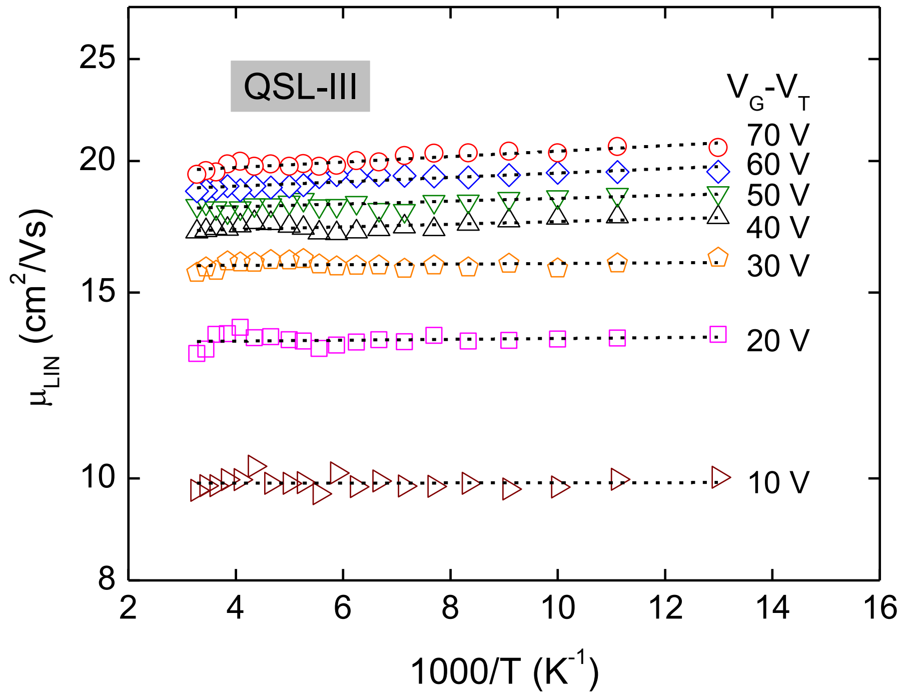

Figure S15. Arrhenius plots of the linear field-effect mobility (μ_{LIN}) calculated at different gate voltages (V_G-V_T) for a QSL-III transistor. High and temperature-independent electron mobility values (negligible E_A; see Table S4) are observed across the entire temperature range investigated. We attribute this to the beneficial role that the Ga_2O_3 interlayer has on the structural and electronic properties of the interface. We note that the electron mobility values calculated for the QSL-III transistors are significantly higher than values extracted for QSL-I transistors. The percolation threshold voltages calculated for the QSL-III transistors (Figure S16) are found to be consistently lower than V_P values calculated for transistors based on QSL-I channels.

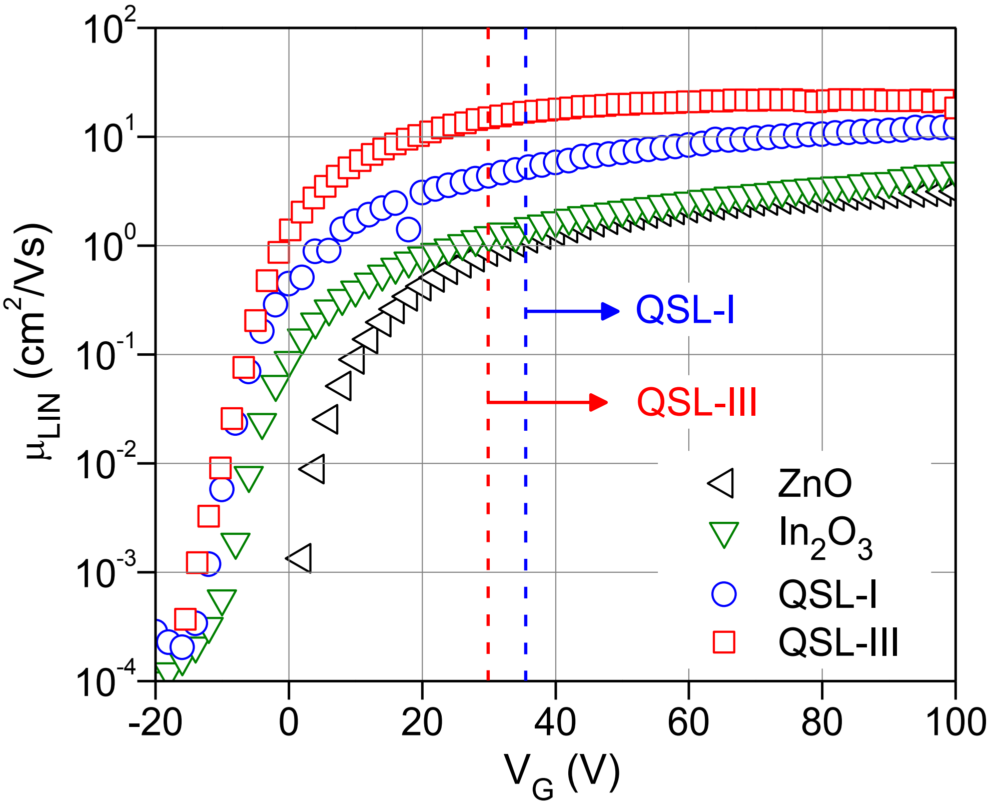

Figure S16. Electron field-effect mobility measured in the linear regime (μ_{LIN}) versus applied gate field (V_G) for the different transistors studied. Using these data the electron transport processes within the channel was further analysed by studying the gate field dependence of electron mobility given as; $\mu_{lin} = K(V_G - V_{T,P})^{\gamma}$ where, V_T, and V_P are threshold voltage and percolation threshold, respectively, with the appropriate chosen values for K and γ.[20] It has been shown that a γ value close to 0.7 would indicate a trap-limited charge (TLC) transport process whilst a value close to 0.1, a percolation dominated conduction (PC) process.[20] Electron transport in single layer transistors is dominated by the TLC mechanism with γ values ~0.95 and ~0.86 for ZnO and In_2O_3, respectively. In contrast, QSL-I and QSL-III transistors show similar characteristics with a clear PC behaviour. The only apparent difference was a lower V_P extracted for the QSL-III transistor.

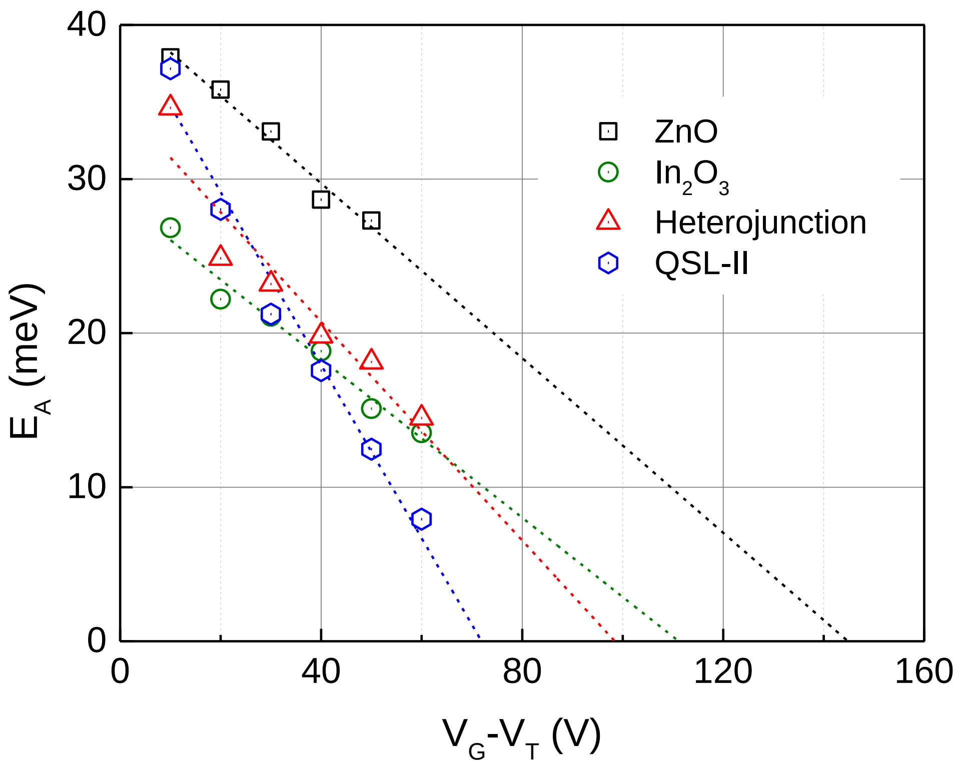

Figure S17. For transistors that do not exhibit percolation conduction behaviour (e.g. ZnO, In_2O_3, heterojunction, and QSL-II based transistors), the V_P can be derived by plotting E_A as a function of (V_G-V_T) and then linearly extrapolating to the intersection at E_A = 0 eV.[18] The V_P values for ZnO, In_2O_3, heterojunction and QSL-II devices can then be obtained yielding 145 V, 110 V, 98 V and 72 V, respectively. A clear improvement in charge transport (indicated by the lower V_P value extracted) can be observed for heterointerface-based transistors as compared to single oxide layer devices. These results provide further direct evidence on the beneficial role that the low-dimensional oxide heterointerfaces play on the overall transistor operation and performance.

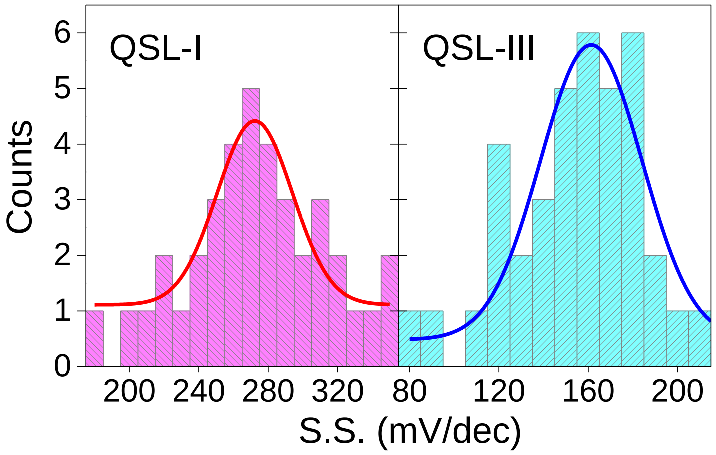

Figure S18. Histogram plots of subthreshold swing (S.S.) measured for the low operating voltage QSL-I and QSL-III based transistors fabricated on glass. The Gaussian fitting curves are guides to the eye.

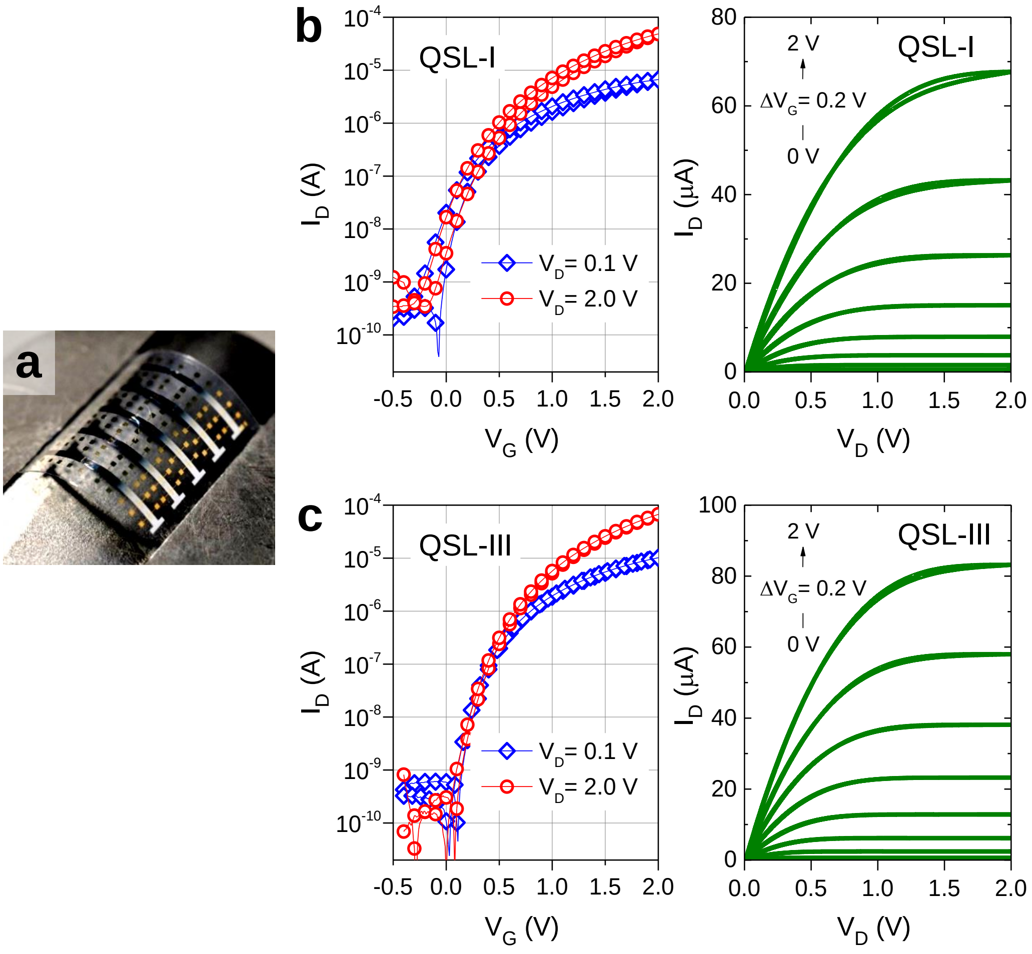

Figure S19. (a) Photograph of an actual low-dimensional oxide QSL-based transistors array containing >35 devices fabricated on a plastic (i.e. PEN) substrate. (b) Representative transfer and output characteristics measured for a QSL-I transistor fabricated on a PEN substrate. (c) Representative transfer and output characteristics measured for a QSL-III transistor fabricated on a plastic substrate.

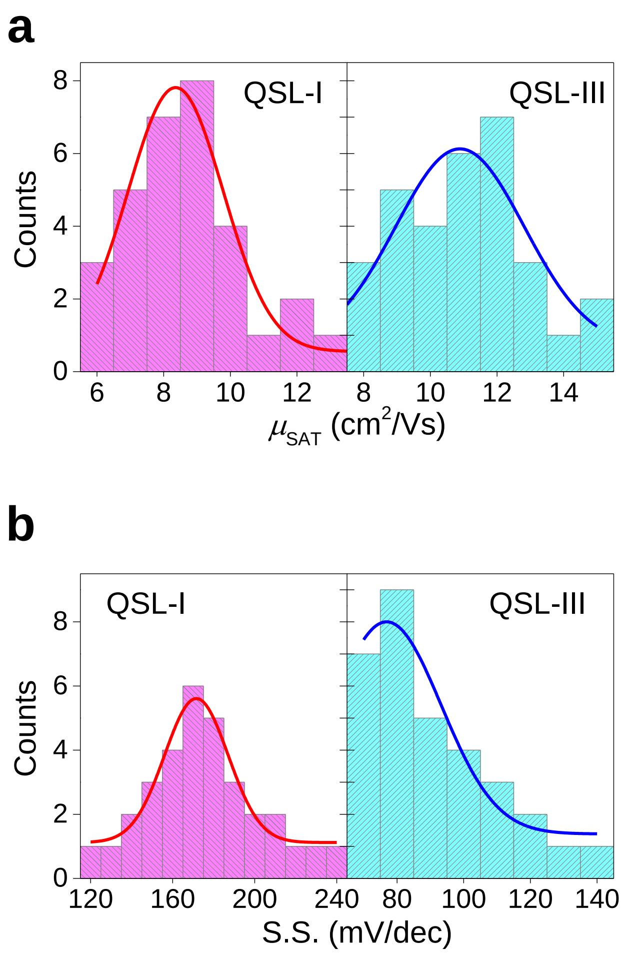

Figure S20. Histogram plots of μ_{SAT} (a) and S.S. swing (b) measured for a large number of low operating voltage oxide QSL-I and QSL-III based transistors fabricated on PEN plastic films. The Gaussian fitting curves are guides to the eye.

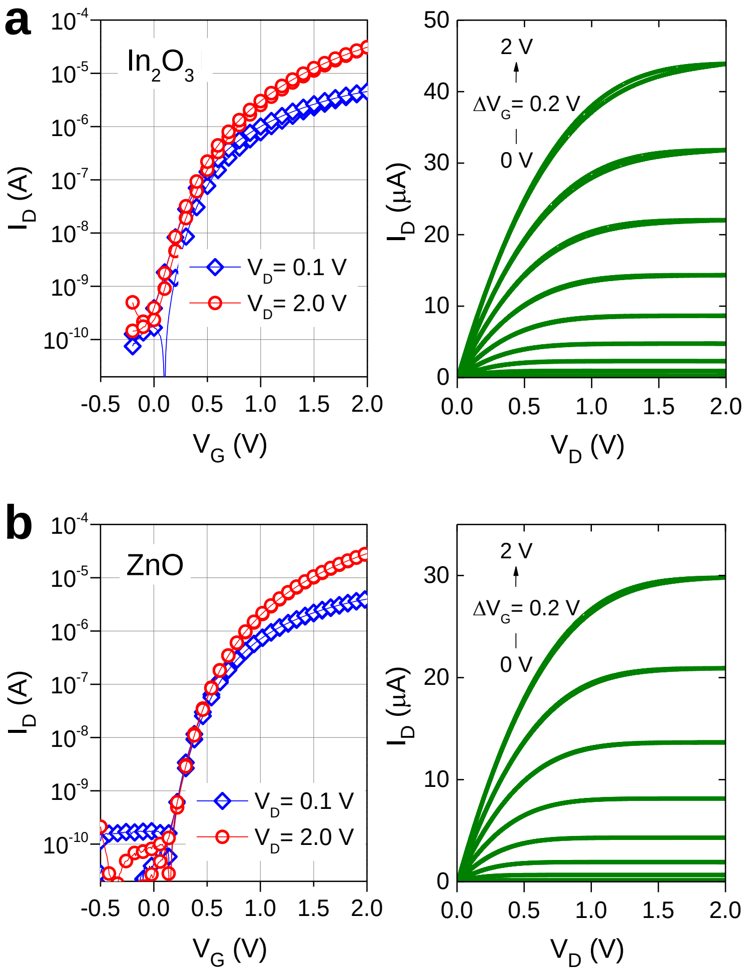

Figure S21. (a) Transfer (left) and output (right) characteristics measured for an In_2O_3-based transistor fabricated on a glass substrate. (b) Transfer (left) and output (right) characteristics measured for a ZnO-based transistor fabricated on a glass substrate.

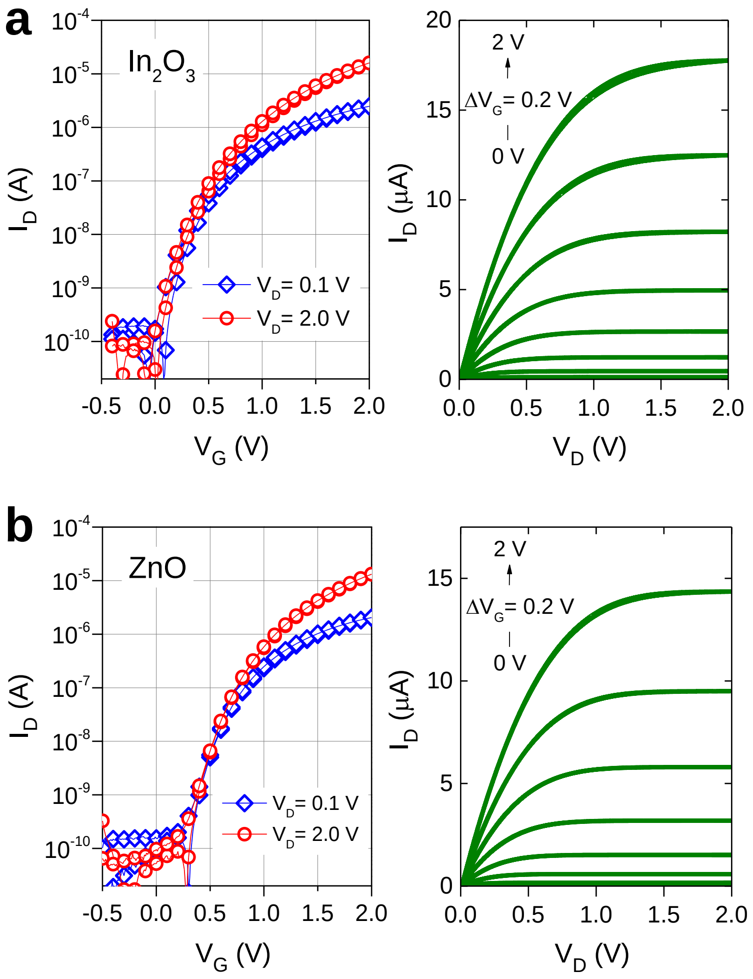

Figure S22. (a) Transfer (left) and output (right) characteristics measured for an In_2O_3-based transistor fabricated on a plastic (i.e. PEN) substrate. (b) Transfer (left) and output (right) characteristics measured for a ZnO-based transistor fabricated on a PEN substrate.

Table S1. Summary of reported electron mobility values* measured for different metal oxide hetero-interface systems grown by different methods.

Metal oxide	μ_{LIN} (cm^2/Vs)	μ_{SAT} (cm^2/Vs)	μ_{Hall} (cm^2/Vs)	Method	T** (°C)	Ref.
QSL***	42.1	46.3	n/a****	Solution	180-200	This work
MgZnO/ZnO or ZnO/ZnMgO	n/a	84.2	n/a	Sputtering	600	[21]
	n/a	n/a	39.4	Sputtering	600	[22]
	5	n/a	n/a	Sputtering	350	[23]
	n/a	9.2	n/a	MOCVD	450	[24]
	n/a	8.9	n/a	MOCVD	450	[25]
	n/a	140	130	MBE	750	[26]
	n/a	80	n/a	MBE	350	[27]
	180	n/a	n/a	MBE	500	[28]
ZnO/Al_2O_3	27.8	n/a	n/a	ALD	200	[29]
ZnO/HZO	6.3	n/a	n/a	ALD	200	[30]

*Electron mobility values measured at room temperature
**Processing temperatures
***Electron mobility calculated from low operation voltage QSL-based transistors made on glass
**** Not available (n/a)

Table S2. Summary of the fitting parameters namely, layer thickness (d), scattering length density (ρ) and interface roughness (R_{rms}), used to fit the experimental XRR spectra for each system studied.

Single metal oxide layers						
**Film**	**ZnO**		**In_2O_3**		**Ga_2O_3**	
**Layer**	ZnO	SiO_2	In_2O_3	SiO_2	Ga_2O_3	SiO_2
**d (Å)**	93.6	N/A	122.04	N/A	35.89	N/A
**ρ ($Å^{-2}$)**	4.50×10^{-5}	1.87×10^{-5}	5.07×10^{-5}	1.87×10^{-5}	4.71×10^{-5}	1.87×10^{-5}
**R_{rms} (Å)**	4.272	11.104	4.66	17.218	3.045	14.217
**Metal oxide bilayers**						
**Film**	**ZnO/ In_2O_3**			**Ga_2O_3/ In_2O_3**		
**Layer**	ZnO	In_2O_3	SiO_2	Ga_2O_3	In_2O_3	SiO_2
**d (Å)**	134.47	85.66	N/A	33.04	116.5	N/A
**ρ ($Å^{-2}$)**	4.50×10^{-5}	5.19×10^{-5}	1.87×10^{-5}	5.60×10^{-5}	4.56×10^{-5}	1.87×10^{-5}
**R_{rms} (Å)**	3.969	15	24.555	3.379	6.5	6.175
**Metal oxide QSL-II**						
**Film**	**ZnO/ Ga_2O_3/In_2O_3**					
**Layer**		ZnO	Ga_2O_3	In_2O_3	SiO_2	
**d (Å)**		60.46	26.73	31.69	N/A	
**ρ ($Å^{-2}$)**		4.50×10^{-5}	4.72×10^{-5}	5.20×10^{-5}	1.87×10^{-5}	
**R_{rms} (Å)**		2.441	2.502	5.3	5.643	

Table S3. Summary of threshold voltage (V_T), turn-on voltage (V_{ON}) and surface charge trap density (N_{tr}) calculated for the different types of transistors studied.[31]

**Device channel**	**V_T (V)**		**V_{ON} (V)**		**N_{tr} (cm^{-2})**	
	293K	77K	293K	77K	293K	77K
**In_2O_3**	5.7	45.6	-13.0	18.9	1.01×10^{12}	1.19×10^{12}
**ZnO**	27.8	18.1	12.0	-4.0	8.48×10^{11}	1.43×10^{12}
**Heterojunction**	9.0	10.9	-6.0	-5.1	8.09×10^{11}	8.62×10^{11}
**QSL-I**	-0.6	4.8	-7.8	-6.5	3.87×10^{11}	6.09×10^{11}
**QSL-II**	5.8	10.0	-3.5	-2.0	4.98×10^{11}	6.47×10^{11}
**QSL-III**	-8.3	5.8	-15.1	-4.2	3.63×10^{11}	5.28×10^{11}

Table S4. Summary of the activation energies (E_A) calculated at different gate voltages (V_G-V_T) for the different types of oxide transistors studied. To be noted that the E_A for the ZnO, In_2O_3, heterojunction, and QSL-II devices was calculated from the high temperature regime (T > 250 K).

		**Thermal activation energy / E_A (meV)**					
		In_2O_3	ZnO	Heterojunction	QSL-I	QSL-II	QSL-III
**V_G-V_T (V)**	10	26.8	37.9	34.6	-4.0	37.2	0
	20	22.2	35.8	24.9	-3.4	28.0	-0.1
	30	21.1	33.1	23.2	-3.0	21.2	-0.1
	40	18.8	28.7	19.8	-2.9	17.6	-0.2
	50	15.1	27.3	18.1	-2.3	12.5	-0.3
	60	13.5	-	14.5	-1.7	7.9	-0.4
	70	-	-	-	-	-	-0.5

References for SI

[1] G. Schön, *J. Electron. Spectrosc. Relat. Phenom.* **1973**, *2*, 75-86.

[2] Y. Mizokawa, H. Iwasaki, R. Nishitani, S. Nakamura, *J. Electron. Spectrosc. Relat. Phenom.* **1978**, *14*, 129-141.

[3] C. C. Surdu-Bob, S. O. Saied, J. L. Sullivan, *Appl. Surf. Sci.* **2001**, *183*, 126-136.

[4] J. I. Jeong, J. H. Moon, J. H. Hong, J. S. Kang, Y. Fukuda, Y. P. Lee, *J. Vac. Sci. Technol. A* **1996**, *14*, 293-298.

[5] T. P. Nguyen, J. Ip, *Appl. Surf. Sci.* **2002**, *195*, 251-262.

[6] E. H. Morales, Y. B. He, M. Vinnichenko, B. Delley, U. Diebold, *New J. Phys.* **2008**, *10*, 125030.

[7] Y. H. Hwang, J. S. Seo, J. M. Yun, H. Park, S. Yang, S. H. K. Park, B. S. Bae, *NPG Asia Mater.* **2013**, *5*, e45.

[8] H. Faber, Y.-H. Lin, S. R. Thomas, K. Zhao, N. Pliatsikas, M. A. McLachlan, A. Amassian, P. A. Patsalas, T. D. Anthopoulos, *ACS Appl. Mater. Interfaces* **2014**, *7*, 782-790.

[9] D. Barreca, A. Gasparotto, C. Maccato, C. Maragno, E. Tondello, *Surf. Sci. Spectra* **2007**, *14*, 19-26.

[10] N. J. Nicholas, G. V. Franks, W. A. Ducker, *Crystengcomm* **2012**, *14*, 1232-1240.

[11] M. Chen, X. Wang, Y. H. Yu, Z. L. Pei, X. D. Bai, C. Sun, R. F. Huang, L. S. Wen, *Appl. Surf. Sci.* **2000**, *158*, 134-140.

[12] Y. Jeong, C. Bae, D. Kim, K. Song, K. Woo, H. Shin, G. Cao, J. Moon, *ACS Appl. Mater. Interfaces* **2010**, *2*, 611-615.

[13] A. Klein, C. Körber, A. Wachau, F. Säuberlich, Y. Gassenbauer, S. P. Harvey, D. E. Proffit, T. O. Mason, *Materials* **2010**, *3*, 4892-4914.

[14] S. R. Thomas, G. Adamopoulos, Y.-H. Lin, H. Faber, L. Sygellou, E. Stratakis, N. Pliatsikas, P. A. Patsalas, T. D. Anthopoulos, *Appl. Phys. Lett.* **2014**, *105*, 092105.

[15] J. G. Labram, Y.-H. Lin, K. Zhao, R. Li, S. R. Thomas, J. Semple, M. Androulidaki, L. Sygellou, M. McLachlan, E. Stratakis, A. Amassian, T. D. Anthopoulos, *Adv. Funct. Mater.* **2015**, DOI: 10.1002/adfm.201403862.

[16] K. Ellmer, A. Klein, B. Rech, *Transparent Conductive Zinc Oxide: Basics and Applications in Thin Film Solar Cells*, Springer, London 2007.

[17] M. Girtan, G. Folcher, *Surf. Coat. Tech.* **2003**, *172*, 242-250.

[18] S. Lee, A. Ahnood, S. Sambandan, A. Madan, A. Nathan, *IEEE Electron Device Lett.* **2012**, *33*, 1006-1008.

[19] Y. Kozuka, A. Tsukazaki, M. Kawasaki, *Appl. Phys. Rev.* **2014**, *1*, 011303.

[20] S. Lee, K. Ghaffarzadeh, A. Nathan, J. Robertson, S. Jeon, C. Kim, I. H. Song, U. I. Chung, *Appl. Phys. Lett.* **2011**, *98*, 203508.

[21] I.-C. Cheng, B.-S. Wang, H.-H. Hou, J.-Z. Chen, *ECS Trans.* **2013**, *50*, 83-93.

[22] H.-A. Chin, I.-C. Cheng, C.-I. Huang, Y.-R. Wu, W.-S. Lu, W.-L. Lee, J. Z. Chen, K.-C. Chiu, T.-S. Lin, *J. Appl. Phys.* **2010**, *108*, 054503.

[23] C.-H. Li, Y.-S. Tsai, J. Z. Chen, *Semicond. Sci. Tech.* **2011**, *26*, 105007.

[24] K. Remashan, Y. S. Choi, S. J. Park, J. H. Jang, *J. Electrochem. Soc.* **2010**, *157*, H1121-H1126.

[25] K. Remashan, Y.-S. Choi, S.-J. Park, J.-H. Jang, *Jpn. J. Appl. Phys.* **2011**, *50*, 04DJ08.

[26] K. Koike, I. Nakashima, K. Hashimoto, S. Sasa, M. Inoue, M. Yano, *Appl. Phys. Lett.* **2005**, *87*, 112106.

[27] S. Sasa, M. Ozaki, K. Koike, M. Yano, M. Inoue, *Appl. Phys. Lett.* **2006**, *89*, 053502.

[28] S. Sasa, T. Hayafuji, M. Kawasaki, K. Koike, M. Yano, M. Inoue, *Jpn. J. Appl. Phys.* **2008**, *47*, 2845.

[29] C. H. Ahn, K. Senthil, H. K. Cho, S. Y. Lee, *Sci. Rep.* **2013**, *3*, 2737.

[30] C. H. Ahn, M. G. Yun, S. Y. Lee, H. K. Cho, *IEEE Trans. Electron Device* **2014**, *61*, 73-78.

[31] Y. H. Lin, H. Faber, S. Rossbauer, T. D. Anthopoulos, *Appl. Phys. Lett.* **2013**, *102*, 193516.
